# Synergistic Ultrasound‐Photo Enhancement of Ferroelectric Catalysis via Molecular Multiferroics

**DOI:** 10.1002/advs.202512878

**Published:** 2025-09-24

**Authors:** Lin‐Yu Zhao, Tai‐Ting Sha, Qiang Pan, Ru‐Jie Zhou, Xiang‐Zhi Zhang, Shi‐Yi Sun, Huihui Hu, Yu‐Meng You

**Affiliations:** ^1^ Jiangsu Key Laboratory for Science and Applications of Molecular Ferroelectrics Southeast University Nanjing 211189 P. R. China

**Keywords:** alkane oxidation, ferroelectric catalytic, molecular multiferroics, photocatalysis, synergistic catalysis

## Abstract

Molecular multiferroics have garnered significant attention for their exceptional responsiveness to external stimuli, demonstrating remarkable potential in multi‐state information storage and sensing technologies. However, their catalytic capabilities remain underexplored. Here, the first demonstration of molecular multiferroic, N‐ethyl‐N‐(fluoromethyl)‐N‐methylethanaminium tetrabromoferrate(III) (DEFM‐FeBr_4_) is presented, achieving efficient catalytic oxidation of alkanes through synergistic multifield activation. Under concurrent ultrasonic and light irradiation, DEFM‐FeBr_4_ exhibits outstanding catalytic performance in the oxidation of ethylmethylbenzene, achieving a turnover number (TON) of 1942—a 29‐fold enhancement in activity compared to the inorganic ferroelectric barium titanate (BaTiO_3_). The material demonstrates excellent recyclability and broad substrate compatibility across diverse alkanes, underscoring its practical advantages in heterogeneous catalysis. Mechanistic investigations via scanning kelvin probe microscopy (SKPM) and electron paramagnetic resonance (EPR) reveal that the intrinsic ferroelectric polarization facilitates the efficient separation of photogenerated charge carriers through built‐in electric field modulation, thereby significantly enhancing catalytic activity. This work not only establishes molecular multiferroics as a new paradigm in catalytic materials science but also provides fundamental insights for designing advanced multiferroic catalysts with optimized multifield‐responsive properties. This multiferroic catalysis strategy opens new avenues for developing smart catalytic systems with dynamically tunable reactivity.

## Graphical abstract

This work demonstrates the first use of molecular multiferroic DEFM‐FeBr_4_ as an efficient catalyst for alkane oxidation using O_2_. Synergistic ultrasound and light activation enable outstanding performance, achieving a TON of 1942—29 times higher than BaTiO_3_. Mechanistic insights reveal ferroelectric polarization enhances charge separation. The approach paves the way for designing advanced multifield‐responsive catalysts.

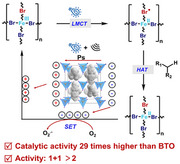

## 1 Introduction

**Figure 1:**
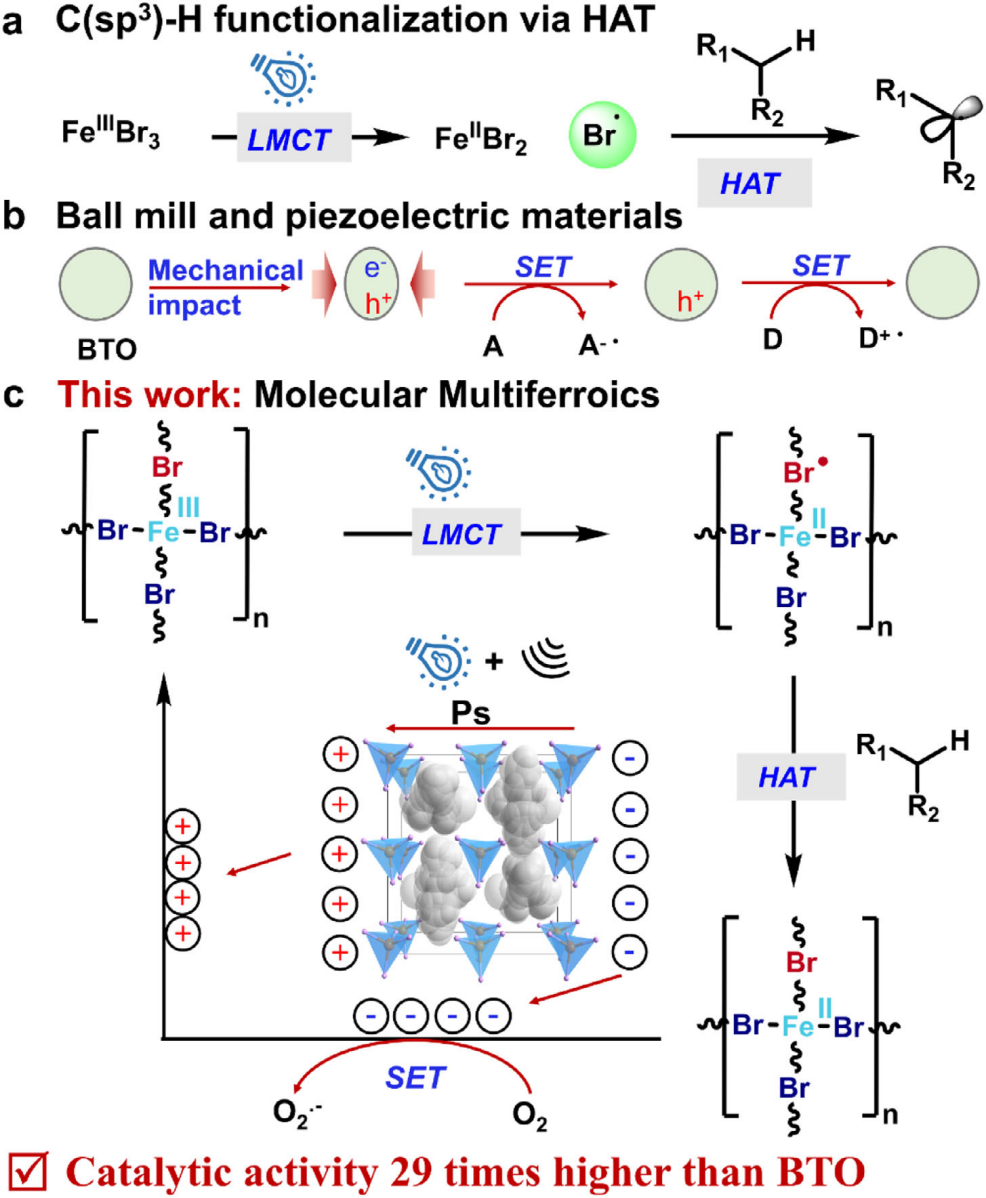
Schematic diagram illustrating the advantages of molecular multiferroics in ferroelectric catalysis. a) Iron‐based catalysts functionalize sp^3^ C─H bonds through hydrogen atom transfer (HAT)^[^ ^7^, ^8^ ^]^; b) Piezoelectric catalysis of inorganic ferroelectrics^[^ ^21^ ^]^; c) Molecular multiferroics for ferroelectric catalysis: achieving 1+1>2, the catalytic activity is 29 times that of BTO (BaTiO_3_).

C─H bond functionalization is a highly efficient and transformative strategy for directly converting ubiquitous C─H bonds in organic molecules into C─C or C─X (e.g., C─O, C─N, C─S) bonds. This approach eliminates the need for substrate prefunctionalization, significantly simplifying organic synthesis pathways and redefining conventional synthetic logic.^[^ ^1^, ^2^, ^3^ ^]^ However, due to the inherent chemical inertness, high bond dissociation energy (BDE), and minimal polarity differences of C─H bonds, achieving efficient and selective C─H activation remains a major challenge.^[^ ^4^, ^5^, ^6^ ^]^ In recent years, radical‐mediated C─H activation has attracted widespread attention for its ability to overcome these obstacles, with photoinduced hydrogen atom transfer (HAT) strategies being particularly prominent^[^ ^7^, ^8^ ^]^ (**Figure** **1**a). For example, ligand‐to‐metal charge transfer (LMCT) processes involving halide anions at transition metal centers can generate halogen radicals, which mediate HAT and enable transformations such as alkylation,^[^ ^9^ ^]^ alkenylation,^[^ ^10^ ^]^ arylation,^[^ ^11^ ^]^ acylation,^[^ ^12^ ^]^ and amination^[^ ^13^ ^]^ of C(sp^3^)─H bonds. Among various transition metal catalysts, 3d transition metals such as Fe,^[^ ^14^ ^]^ Cu,^[^ ^15^ ^]^ and Ni^[^ ^16^ ^]^ have garnered significant interest due to their earth abundance, cost‐effectiveness, and tunable reactivity.

Ferroelectric catalysis is an emerging strategy that leverages the spontaneous polarization properties of ferroelectric materials to enhance catalytic performance.^[^ ^17^ ^]^ The polarization direction of ferroelectric materials can be dynamically regulated by external stimuli (e.g., pressure, temperature, light, or ultrasound), thereby altering internal electric fields and surface charge distributions to promote charge separation. Additionally, ferroelectric polarization can modulate the thermodynamic stability of surface chemistry, influencing adsorption and desorption processes to improve catalytic efficiency and selectivity. Furthermore, the reversible polarization switching at domain walls can suppress undesired side reactions, enhancing product selectivity. In recent years, inorganic ferroelectric materials have demonstrated great potential in photocatalytic water splitting,^[^ ^18^ ^]^ dye degradation,^[^ ^19^ ^]^ CO_2_ reduction,^[^ ^20^ ^]^ and organic synthesis^[^ ^21^ ^]^ (Figure 1b). For instance, BaTiO_3_ has attracted significant attention due to its unique ferroelectric properties and high catalytic activity, which can be further optimized through morphology control, composite material design, and exposure of highly active crystal facets.^[^ ^22^, ^23^, ^24^, ^25^ ^]^ However, complex catalytic systems such as C−H activation, asymmetric catalysis, and hydrogenation/dehydrogenation often require specific metal active sites, which are difficult to introduce into inorganic ferroelectric materials through minor doping. Excessive doping, on the other hand, may disrupt the crystal structure and lead to the loss of ferroelectric properties, limiting their widespread application in catalysis. Additionally, under multiple external stimuli, inorganic ferroelectric materials tend to undergo structural instability and deactivation, further reducing their practical catalytic value.

Molecular ferroelectric materials,^[^ ^26^ ^]^ as a crucial complement to inorganic ferroelectrics, offer exceptional tunability and structural diversity, providing new possibilities for catalysis. While molecular ferroelectric materials have demonstrated immense potential in sensing, energy storage, and semiconductor applications, their catalytic properties remain largely unexplored. Through rational molecular design and functional group modifications, molecular ferroelectric materials can achieve precise control over catalytic activity, facilitating highly efficient and selective transformations. Moreover, the structural versatility of organic molecules allows them to adapt to diverse catalytic environments, creating unprecedented opportunities for exploring new catalytic mechanisms and optimizing reaction conditions. Multiferroic materials,^[^ ^27^, ^28^ ^]^ which simultaneously exhibit ferroelectricity, ferromagnetism, and ferroelasticity in a single phase, can undergo reversible regulation of polarization, magnetization, or strain under external stimuli such as electric fields, magnetic fields, or mechanical stress. Owing to these unique physical properties, multiferroic materials have been widely applied in data storage, sensors, transducers, and energy harvesting. However, the number of known multiferroic materials remains limited, and their catalytic potential, particularly in multi‐field catalysis, remains largely unexplored.

In this study, we pioneer the application of a molecular multiferroic material—N‐ethyl‐N‐(fluoromethyl)‐N‐methylethanaminium tetrabromoferrate(III) (DEFM‐FeBr_4_)—for alkane oxidation catalysis (Figure 1c). Under dual ultrasound‐light activation, DEFM‐FeBr_4_ exhibits significantly superior catalytic activity compared to inorganic ferroelectric and piezoelectric materials (e.g., BaTiO_3_), achieving a 29‐fold increase in activity. Additionally, we systematically investigate the effect of ultrasonic power on catalytic activity and explore the role of molecular multiferroic materials in the C─H activation process. Notably, this study examines C─H activation reactions from the perspective of molecular ferroelectric catalysis, highlighting the immense potential of molecular multiferroic materials as innovative catalysts. A deeper understanding of selective C─H oxidation reactions under multi‐field synergy not only promotes the development of sustainable and efficient organic synthesis but also provides new insights for the rational design of next‐generation high‐performance ferroelectric catalysts.

## 2 Results and Discussion

### 2.1 Synthesis and Characterization

**Figure 2:**
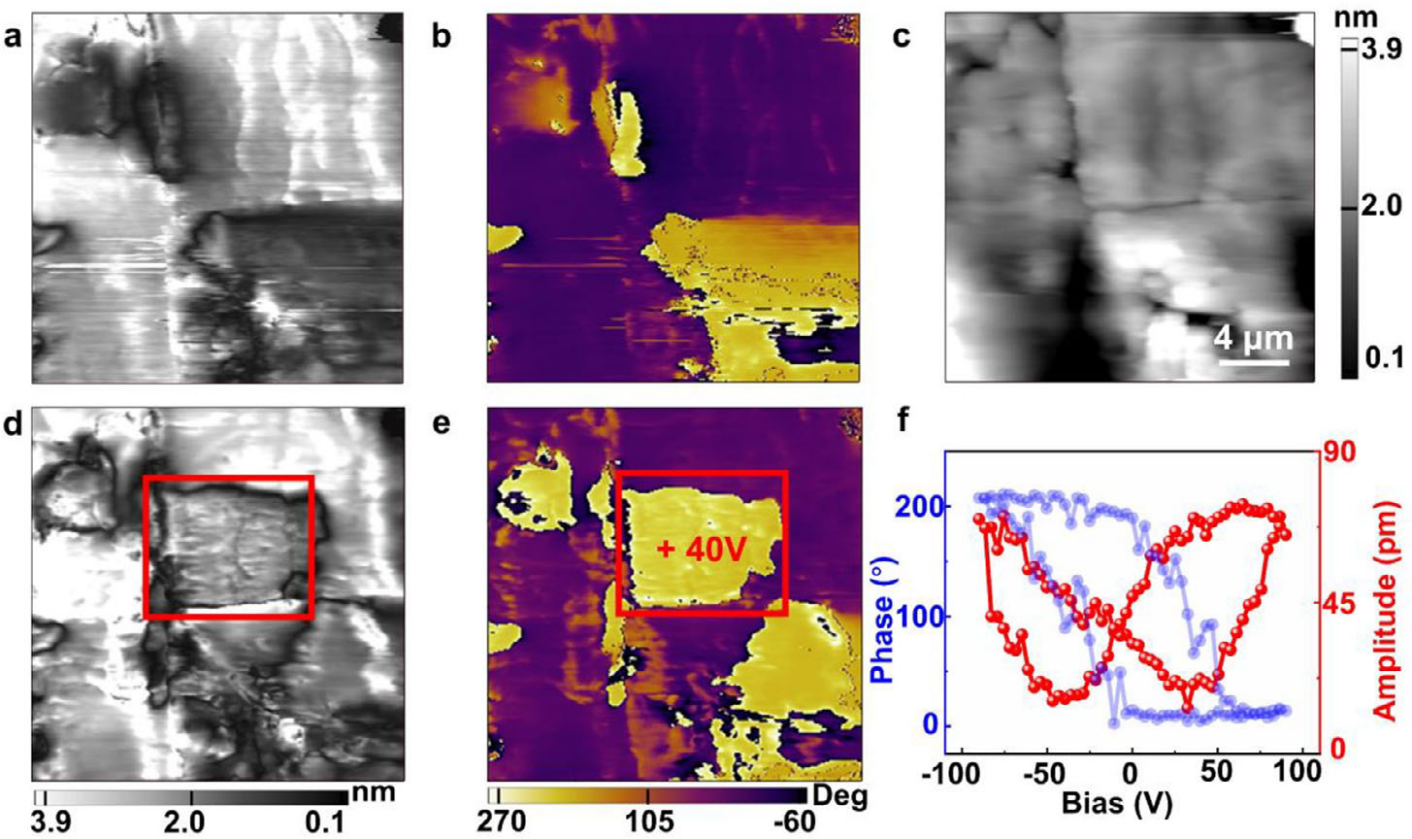
Ferroelectric characterization of DEFM‐FeBr_4_. a) Initial PFM amplitude, b) PFM phase, and c) topographic images. d) PFM amplitude and e) phase images after applying a voltage of +40 V on the red‐boxed region. f) The phase hysteresis loop and butterfly‐shaped amplitude curve.

We successfully synthesized the molecular multiferroic material N‐ethyl‐N‐(fluoromethyl)‐N‐methylethanaminium tetrabromoferrate(III) (DEFM‐FeBr_4_) following a reported method.^[^ ^29^ ^]^ Powder X‐ray diffraction (PXRD) analysis confirmed the successful synthesis of the compound with high purity and excellent crystallinity (Figure S1, Supporting Information). Additionally, we characterized the ferroelectric domain structure of DEFM‐FeBr_4_ thin films using piezoresponse force microscopy (PFM). Both the PFM phase and amplitude images clearly revealed the presence of ferroelectric domains, which are consistent with previously reported results (**Figure** **2**). To further investigate the ferroelectric properties of this material, we conducted PFM dynamic domain switching experiments, enabling direct visualization of domain evolution under an applied electric field. Initially, the selected region exhibited a multidomain structure, which underwent directional polarization switching upon the application of a +40 V voltage (Figure 2a–e), further validating the ferroelectric nature of DEFM‐FeBr_4_. Moreover, to confirm the switchable polarization behavior under an external electric field, we applied a ±80 V scanning voltage on the thin film surface (Figure 2f), effectively distinguishing the local polarization directions from the piezoresponse intensity. The domain walls were clearly visible in the amplitude images, and these signals were independent of the simultaneously acquired surface topography data (Figure 2c), ensuring the accuracy and reliability of the analysis. Collectively, these results provide strong evidence for the successful synthesis of the molecular multiferroic material DEFM‐FeBr_4_ with tunable polarization characteristics.

### 2.2 Ferroelectric Catalysis

**Figure 3:**
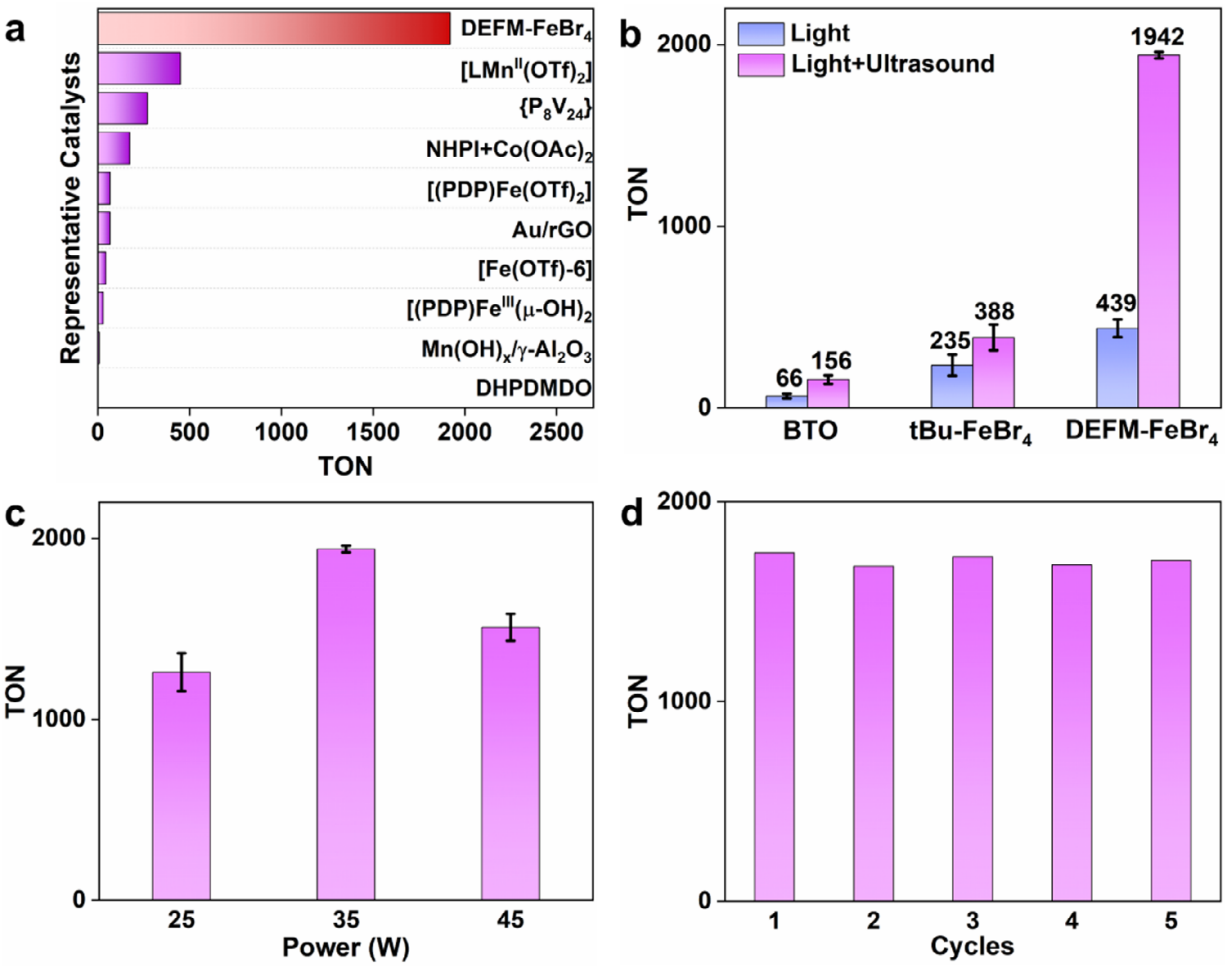
Ferroelectric catalytic performance. a) Comparison of the catalytic activity for the oxidation of 4‐ethyltoluene to 4′‐methylacetophenone using DEFM‐FeBr_4_ and other catalysts. b) TON for the oxidation of 4‐ethyltoluene catalyzed by BTO, tBu‐FeBr_4_, and DEFM‐FeBr_4_ under 365 nm LED irradiation (370 mW cm^−2^) and/or ultrasound (35 W, 20 kHz) conditions for 24 h. c) TON of 4‐ethyltoluene oxidation catalyzed by DEFM‐FeBr_4_ under varying ultrasonic power (25, 35, and 45 W) with 365 nm LED irradiation (370 mW cm^−2^) for 24 h. d) Five consecutive cycles of DEFM‐FeBr_4_ for the oxidation of 4‐ethyltoluene under 365 nm LED (370 mW cm^−2^) and ultrasound (35 W, 20 kHz).

We systematically investigated the catalytic performance of the molecular multiferroic material DEFM‐FeBr_4_ in the oxidation of 4‐ethyltoluene to 4′‐methylacetophenone using O_2_ as the oxidant. Under optimized conditions, 4‐ethyltoluene was dissolved in hexafluorobenzene containing suspended DEFM‐FeBr_4_, and the reaction was conducted under 365 nm LED irradiation combined with ultrasonic treatment for 24 h. The turnover number (TON) for 4′‐methyl acetophenone reached 1942, significantly surpassing most reported catalytic oxidation systems for 4‐ethyltoluene^[^ ^30^, ^31^, ^32^, ^33^, ^34^, ^35^, ^36^, ^37^ ^]^ (**Figure** **3**a). To explore the importance of the synergistic effects of multiple external fields, we conducted control experiments. The results showed that in the absence of light but with ultrasound, the catalytic activity was nearly negligible (Figure S2, Supporting Information). In contrast, under light irradiation without ultrasound, the TON was only 439 (Figure 3b). This clearly indicates that both the optical and mechanical fields are indispensable and jointly contribute to the enhancement of catalytic activity. Furthermore, we compared the catalytic performance of DEFM‐FeBr_4_ and the inorganic ferroelectric material BaTiO_3_ (BTO) under identical ultrasonic conditions (Figure 3b). The results demonstrated that the catalytic activity of DEFM‐FeBr_4_ (TON = 1942) was 29 times higher than that of BTO (TON = 66), further confirming the great potential of molecular multiferroic materials in catalysis. We attribute this remarkable activity difference to several factors: unlike the structurally rigid inorganic ferroelectric materials, molecular multiferroic materials have lower acoustic impedance and better compatibility with solvents. More importantly, molecular multiferroic materials possess a higher density of metal active sites, and their ferroelectric polarization typically occurs near the catalytic active sites. The built‐in electric field can directly modulate catalytic reactions and enhance charge separation efficiency, thereby significantly improving catalytic efficiency.

To further verify the role of ferroelectricity in catalysis, we synthesized a non‐ferroelectric material, tetrabutylammonium iron bromide (tBu‐FeBr_4_), following a reported method (Figure S3, Supporting Information),^[^ ^38^ ^]^ and evaluated its catalytic activity in the oxidation of 4‐ethyltoluene to 4′‐methyl acetophenone. The results showed that under identical reaction conditions, the TON for tBu‐FeBr_4_ was 388, whereas that for DEFM‐FeBr_4_ was 1942, representing a 5‐fold increase in catalytic activity (Figure 3b, Figures S4 and S5, Supporting Information). This significant enhancement further confirms the crucial role of ferroelectricity in improving catalytic performance. We believe this improvement is primarily due to the spontaneous polarization and built‐in electric field effects of DEFM‐FeBr_4_, which effectively promote the separation of photogenerated electron‐hole pairs, reduce charge recombination, and thereby enhance photocatalytic efficiency. Additionally, the internal electric fields in ferroelectric materials facilitate directional charge carrier migration, while ultrasonic waves induce reorientation of molecular dipoles, dynamically modulating the built‐in electric field and altering surface charge distribution, further boosting the catalytic activity of DEFM‐FeBr_4_.

We also explored the influence of ultrasonic power on catalytic activity (Figure 3c). Using 4‐ethyltoluene as the substrate, we observed a steady enhancement in catalytic performance as the ultrasonic power increased from 25 to 35 W. This improvement can be attributed to the higher ultrasonic power promoting the reorientation of ferroelectric dipoles within DEFM‐FeBr_4_, thereby strengthening the built‐in electric field and facilitating charge separation. However, further increasing the power to 45 W resulted in reduced activity, likely due to catalyst destabilization at excessive power levels. These results emphasize the importance of optimizing ultrasonic parameters to maximize catalytic output.

Finally, we evaluated the cycling stability of DEFM‐FeBr_4_ (Figure 3d). Over five consecutive catalytic cycles, the catalyst maintained excellent catalytic activity, demonstrating outstanding stability. Furthermore, post‐reaction characterization through PXRD analysis confirmed the structural integrity of DEFM‐FeBr_4_ (Figure S6, Supporting Information), and temperature monitoring experiments were conducted to rule out any thermal effects on the catalytic results (Figure S7, Supporting Information). These findings indicate that DEFM‐FeBr_4_, as a molecular multiferroic heterogeneous catalyst, holds great promise for applications in multi‐field synergistic catalytic oxidation reactions.

Subsequently, under the optimized reaction conditions, we evaluated the substrate scope of this catalytic system using DEFM‐FeBr_4_ as the catalyst (**Figure** **4**). 4‐Ethyltoluene and various functionalized substrates containing different sp^3^ C─H bonds were successfully converted into the corresponding ketone products. The catalytic efficiency varied depending on the reactivity of the C─H bonds in different substrates. Notably, α‐position compounds with benzylic C─H bonds were efficiently oxidized to the desired ketone products, highlighting the system's high selectivity and effectiveness (Figures S8–23, Supporting Information).

**Figure 4:**
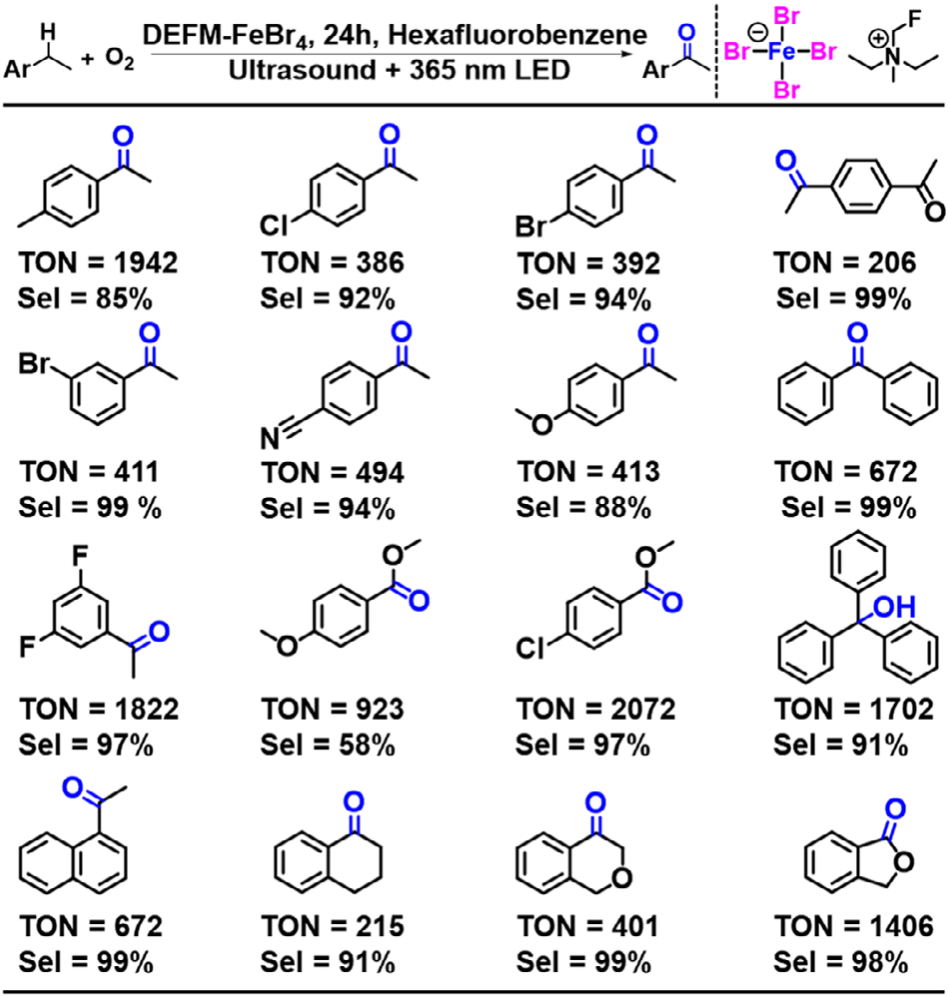
Substrate scope of DEFM‐FeBr_4_ in ferroelectric‐catalyzed C─H oxidation. Reactions were carried out using 0.4 mL of substrate in 4 mL of hexafluorobenzene under 365 nm LED irradiation (370 mW cm^−2^) and ultrasound (35 W, 20 kHz) for 24 h. Turnover numbers (TONs) and product selectivity were determined by GC‐MS and ^1^H‐NMR analysis.

### 2.3 Mechanism of Ferroelectric Catalysis

**Figure 5:**
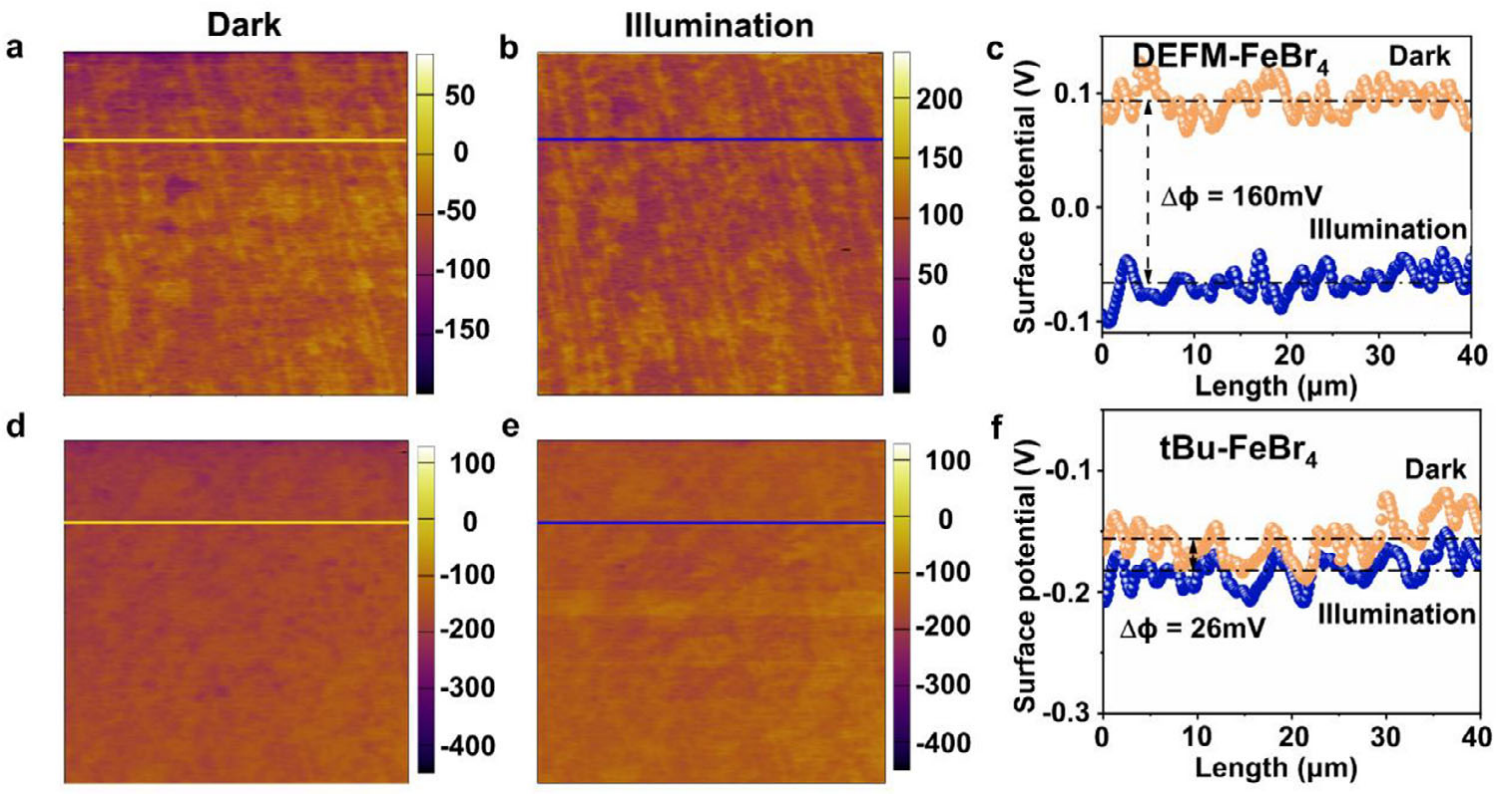
Scanning Kelvin Probe Microscopy (SKPM) potential imaging of DEFM‐FeBr_4_ and tBu‐FeBr_4_. a,d) Surface potential images of DEFM‐FeBr_4_ (a) and tBu‐FeBr_4_ (d) measured under dark conditions, highlighting the baseline electrostatic potential; b,e) Surface potential images of DEFM‐FeBr_4_ (b) and tBu‐FeBr_4_ (e) measured under illumination, demonstrating the changes induced by light exposure; c,f) Comparative surface potential profiles of DEFM‐FeBr_4_ (c) and tBu‐FeBr_4_ (f) measured at the same locations under both dark and illuminated conditions.

To further deepen our understanding of the observed catalytic behavior, we utilized Scanning Kelvin Probe Microscopy (SKPM) to observe the changes in surface potential of the molecular multiferroic material DEFM‐FeBr_4_ (**Figure** **5**) and the non‐ferroelectric material tBu‐FeBr_4_ before and after light irradiation. Interestingly, under light irradiation, the surface potential of DEFM‐FeBr_4_ decreased by 160 mV compared to its dark state (Figure 5a–c). This phenomenon may be related to the influence of its built‐in electric field, which causes photo‐generated electrons to migrate to the surface and be partially depleted by the surface polarized charges. This indicates that the built‐in electric field within the catalyst effectively promotes the separation of photo‐generated charge carriers. In contrast, the non‐ferroelectric material tBu‐FeBr_4_ showed a small change in surface potential under light irradiation (a decrease of 26 mV compared to the dark state) (Figure 5d–f), indicating that no significant charge imbalance occurred on its surface, which is consistent with its lower catalytic activity. Through the analysis of the surface potential changes, we further demonstrate that the built‐in electric field of the molecular multiferroic material DEFM‐FeBr_4_ plays an important role in the photocatalytic process, effectively promoting the separation of photo‐generated charge carriers, thus enhancing catalytic activity. The non‐ferroelectric material tBu‐FeBr_4_, however, did not generate a noticeable charge separation effect, leading to its lower catalytic activity. These results highlight the crucial role of the built‐in electric field in photocatalytic reactions.

Next, based on relevant literature reports,^[^ ^39^ ^]^ we hypothesize that the catalytic process of DEFM‐Fe^III^Br_4_ is initiated by ligand‐to‐metal charge transfer (LMCT) under light irradiation, resulting in the formation of bromine radicals (Br^•^). These Br^•^ species serve as efficient hydrogen atom transfer (HAT) agents capable of cleaving various C(sp^3^)‐H bonds (Figure S24, Supporting Information). The photoexcited LMCT process initially generates a reactive intermediate, DEFM‐Br^•^‐Fe^II^Br_3_, which subsequently generates alkyl free radicals via the classical HAT process. To verify the involvement of alkyl radicals, the radical scavenger 2,2,6,6‐tetramethylpiperidin‐1‐oxyl (TEMPO) was introduced into the reaction system. As anticipated, only trace amounts of the target product were detected, while the formation of TEMPO–alkyl adducts was confirmed by liquid chromatography–mass spectrometry, thereby substantiating the generation of alkyl radicals during the reaction (Figure S25, Supporting Information). Following this step, the reduced intermediate DEFM‐Fe^II^Br_4_ reacts with O_2_ to produce superoxide radicals (O_2_^•−^), completing the catalytic cycle. To further confirm the formation of O_2_^•−^, electron paramagnetic resonance (EPR) spectroscopy was performed using 5,5‐dimethyl‐1‐pyrroline N‐oxide (DMPO) as a radical trap. Under illumination conditions, DEFM‐FeBr_4_ yielded a weak DMPO─OOH signal; however, upon the addition of ultrasound, a pronounced DMPO─OOH signal was observed (Figure S26, Supporting Information), indicating the enhanced generation of O_2_^•−^. These findings collectively highlight the role of the molecular multiferroic material in promoting charge separation and facilitating the formation of reactive oxygen species, thereby significantly enhancing the catalytic efficiency.

## 3 Conclusion

In conclusion, this study strategically employs molecular multiferroic materials to establish a novel catalytic system for alkane oxidation using O_2_ as a sustainable oxidant. The molecular multiferroic catalyst DEFM‐FeBr_4_, activated by the synergistic effects of ultrasound and light, exhibits outstanding catalytic performance, achieving a high turnover number (TON = 1942) in the oxidation of 4‐ethyltoluene. Compared to traditional inorganic ferroelectrics such as BTO, the catalytic activity is enhanced 29‐fold. This remarkable improvement highlights the unique advantages of molecular multiferroic materials over rigid inorganic counterparts, particularly due to the precise synergy between ferroelectric polarization and transition metal reactivity. The modular nature of molecular ferroelectrics enables rational design of catalytic functions through targeted molecular engineering, offering unprecedented opportunities to expand reaction diversity and push beyond the performance limits of inorganic systems.

## Conflict of Interest

The authors declare no conflict of interest.

## Supporting information



Supporting Information

## Data Availability

The data that support the findings of this study are available in the supplementary material of this article.
