# Supplementary material for: Synergistic Ultrasound‐Photo Enhancement of Ferroelectric Catalysis via Molecular Multiferroics
Source: Adv Sci (Weinh). 2025 Sep 24;12(46):e12878. doi: 10.1002/advs.202512878 (PMC12697854; doi:10.1002/advs.202512878)
Supplement: Supplementary file 1 — Supporting Information [file ADVS-12-e12878-s001.docx]

Synergistic Ultrasound-Photo Enhancement of Ferroelectric Catalysis via Molecular Multiferroics

Lin-Yu Zhao, Tai-Ting Sha, Qiang Pan, Ru-Jie Zhou, Xiang-Zhi Zhang, Shi-Yi Sun, Huihui Hu*, Yu-Meng You*

Jiangsu Key Laboratory for Science and Applications of Molecular Ferroelectrics, Southeast University, Nanjing 211189, P. R. China.

*Correspondence to: huhuihui@seu.edu.cn; youyumeng@seu.edu.cn.

Table of Contents

[1. General Experimental and Chemicals 2](#_Toc191934774)

[2. Characterization of catalysis 3](#_Toc191934775)

[3. ^1^H NMR spectrum of the products 5](#_Toc191934776)

# 1. General Experimental and Chemicals

**General experimental.** Reagents were commercially available and used without further purification unless otherwise indicated. The powder X-ray diffraction (PXRD) data were collected on Rigaku D/MAX 2000 PC X-ray diffraction system with Cu Kα radiation in the 2θ range of 5°–50° with a step size of 5°.

The PFM measurement was carried out on a commercial piezoresponse force microscope (Oxford instrument, MFP-3D) with high-voltage package and in-situ heating stage. PFM is based on the atomic force microscopy (AFM), with an AC drive voltage applied to the conductive tip. Conductive Pt/Ir-coated silicon probes (EFM, Nanoworld) were used for domain imaging and polarization switching studies, with a nominal spring constant of ~2.8 nN/nm and a free-air resonance frequency of ~75 kHz. Since the amplitude of the low-frequency vertical PFM was within the noise level of the quadrant photodetector of the AFM, we performed the PFM experiments at contact resonance. The typical drive frequency was in the range of 320 to 380 kHz for out-of-plane PFM images and 660 to 780 kHz for in-plane PFM images, depending on the contact resonant frequency.

Gas chromatography-mass spectrometry (GCMS) measurement was carried out on Shimadzu QP2020 NX with HP-5MS column.

Synthesis of DEFM-FeBr_4_: An equimolar amount of diethylmethylamine (200 mmol) and bromofluoromethane (200 mmol) was stirred in 200 mL of ethanol at room temperature for 72 hours. After completion of the reaction, the solvent was removed under reduced pressure, yielding a colorless to pale yellow solid sample of N-ethyl-N-(fluoromethyl)-N-methylethylammonium bromide. Subsequently, in a 200 mL beaker, a stoichiometric amount of N-ethyl-N-(fluoromethyl)-N-methylethylammonium bromide and FeBr₃ (20 mmol) was dissolved in 150 mL of deionized water to form a clear solution. After approximately two weeks of slow evaporation at room temperature, dark red to black (DEFM)FeBr₄ crystals were obtained.

General procedure for ferroelectric catalysis experiment: For the ferroelectric-catalyzed oxidation of alkanes, DEFM-FeBr_4_ (1 μmol) was placed in an 8 mL septum-sealed glass vial containing 4 mL of hexafluorobenzene. Next, 400 μL of the substrate was added to the mixture. The probe of a 20 kHz ultrasonic cell disruptor was carefully positioned in the solution, and the ultrasonic power was set to 35 W. Simultaneously, the reaction mixture was irradiated with a 365 nm LED light source at an intensity of 370 mW·cm^-2^, measured at the surface of the reaction mixture using a Thorlabs PM100D (S120VC sensor) photometer. The reaction was carried out for 24 hours under ultrasound and light. After completion, the solid catalyst was separated by centrifugation, and the supernatant was analyzed by GC-MS and ^1^H NMR.

General Procedure of Ferroelectric Catalysis for 4-Ethyltoluene Oxidation: In a typical procedure, DEFM-FeBr_4_ (1 μmol, corresponding to 1.0 μmol Fe active sites, molecular formula C_6_H_15_Br_4_FFeN) was placed in an 8 mL septum-sealed glass vial containing 4 mL of hexafluorobenzene. Subsequently, 400 μL of 4-ethyltoluene (2.87 mmol, density = 0.867 g/mL, substrate-to-catalyst molar ratio ≈ 2870:1) was added to the mixture. The probe of an ultrasonic cell disruptor was positioned appropriately within the solution. The reaction was carried out for 24 h under simultaneous ultrasonication (35 W, 20 kHz) and UV-light irradiation (365 nm LEDs, 370 mW·cm^-2^; intensity measured with a calibrated photometer, Thorlabs PM100D with S120VC sensor). Upon completion, the solid catalyst was recovered by centrifugation, and the supernatant was analyzed using GC-MS and ^1^H-NMR spectroscopy. Yields were calculated based on GC-MS calibration curves with an internal standard.

Gram-scale synthesis: DEFM-FeBr_4_ (8 μmol), used for ferroelectric catalytic alkane oxidation, was placed in a 40 mL septum-sealed glass vial with 22 mL of hexafluorobenzene. Then, 8 mL of 4-ethyltoluene was added to the mixture. Then, insert the probe part of the cell crusher into the appropriate position of the solution. The probe of an ultrasonic cell disruptor was positioned appropriately within the solution. The reaction was carried out for 36 h under simultaneous ultrasonication (45 W, 20 kHz) and UV-light irradiation (365 nm LEDs, 370 mW·cm^-2^; intensity measured with a calibrated photometer, Thorlabs PM100D with S120VC sensor). Upon completion, the solid catalyst was recovered by centrifugation, and the supernatant was analyzed using GC-MS. After analysis, 1.23 g of 4’-methylacetophenone was obtained, with a TON of 1139 and a selectivity of 83%, further demonstrating the practicality and scalability of the catalytic system.

# 2. Characterization of catalysis





**Fig. S1.** The measured powder X-ray diffraction pattern of DEFM-FeBr_4_ at 293 K and the comparison with the simulation of the crystal data.


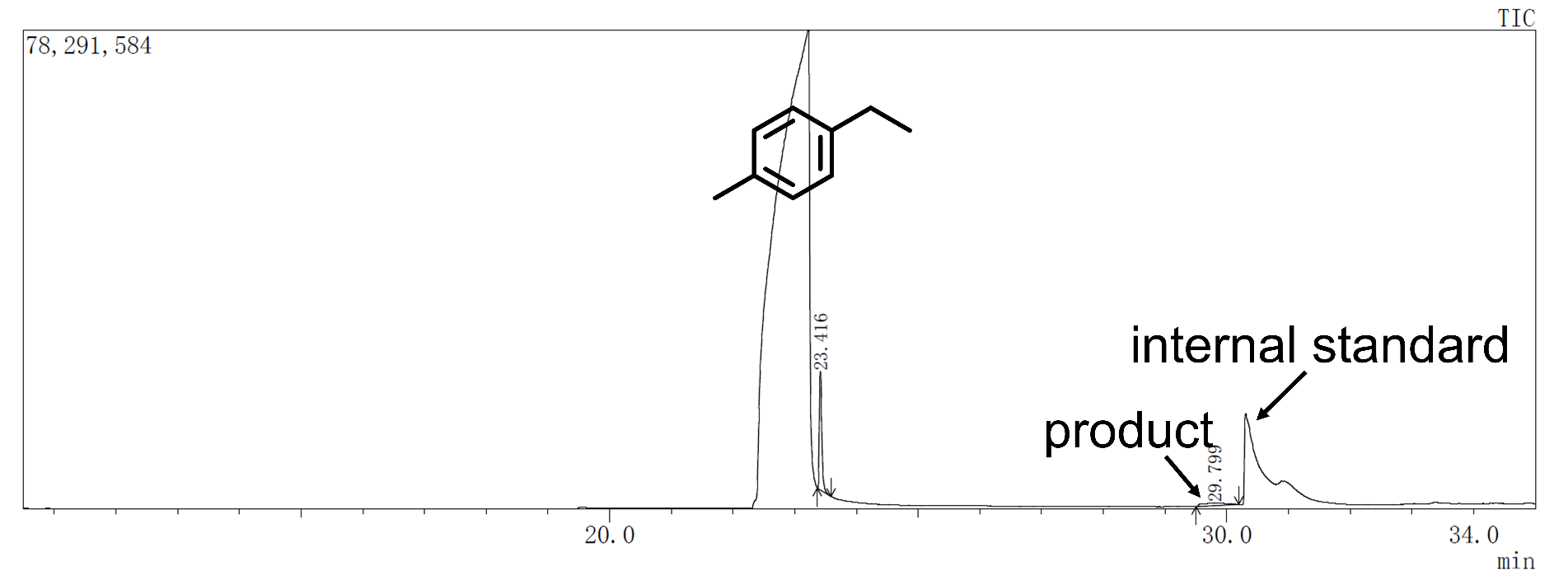


**Fig. S2.** GC-MS trace of the reaction of 4-ethyltoluene under dark reaction conditions without irradiation.





**Fig. S3.** The measured powder X-ray diffraction pattern of tBu-FeBr_4_ at 293 K and the comparison with the simulation of the crystal data.





**Fig. S4.** The measured powder X-ray diffraction pattern of BiFeO_3_ at 293 K and the comparison with the simulation of the crystal data. The synthesis of BiFeO_3_ was carried out following a previously reported literature method (Nanoscale, *2016*, **8**, 7343).





**Fig. S5.** TON for the oxidation of 4-ethyltoluene catalyzed by BTO, PbTiO_3_, BiFeO_3_, KH_2_PO_4_ and DEFM-FeBr_4_ under 365 nm LED irradiation (370 mW·cm^-2^) and/or ultrasound (35 W, 20 kHz) conditions for 24 h.





**Fig. S6.** PXRD patterns of DEFM-FeBr_4_ before and after reaction.


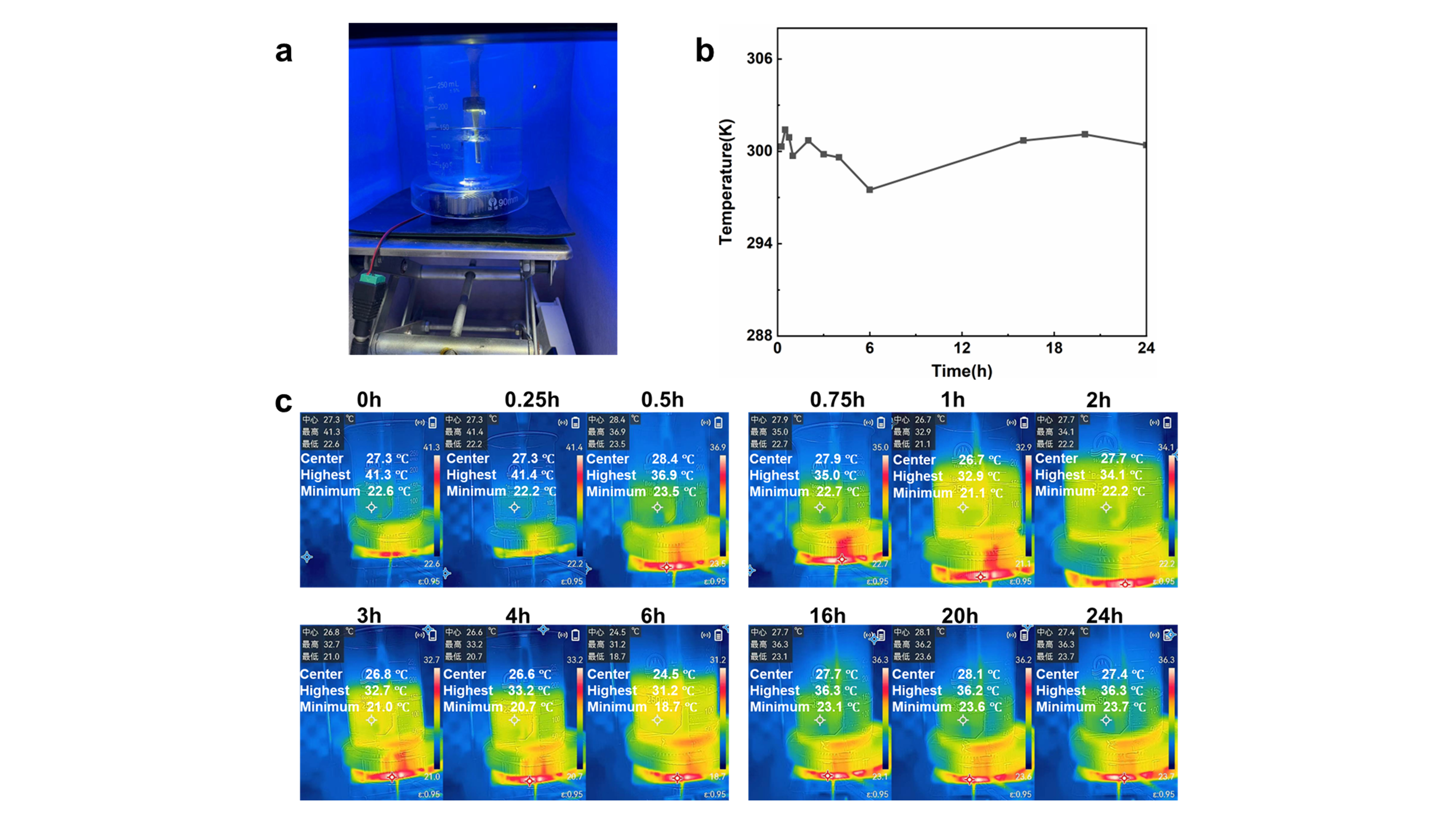


**Fig. S7.** (a) Schematic illustration of the reaction setup with an external water bath for heat dissipation. (b) Time-dependent temperature profile of the reaction system under ultrasonic irradiation, showing stable conditions within ±2 K. (c) Representative time-resolved infrared thermography images recorded by a Hikmicro H21 Pro handheld camera, confirming the absence of significant ultrasound-induced heating.

# 3. ^1^H NMR spectrum of the products


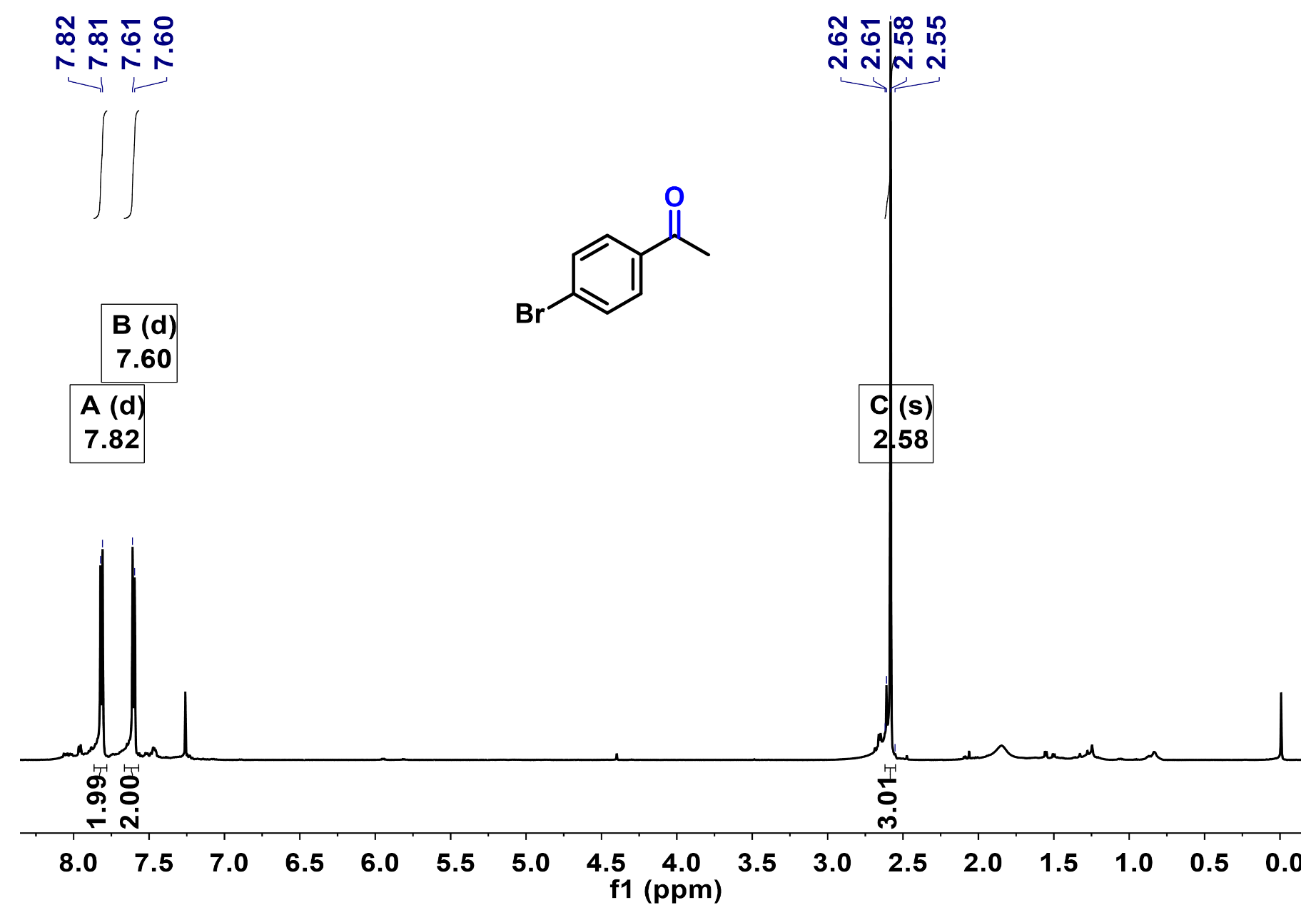


**Fig. S8.** ^1^H NMR spectrum (600 MHz, CDCl_3_) of 4'-bromoacetophenone after TLC separation for purification.


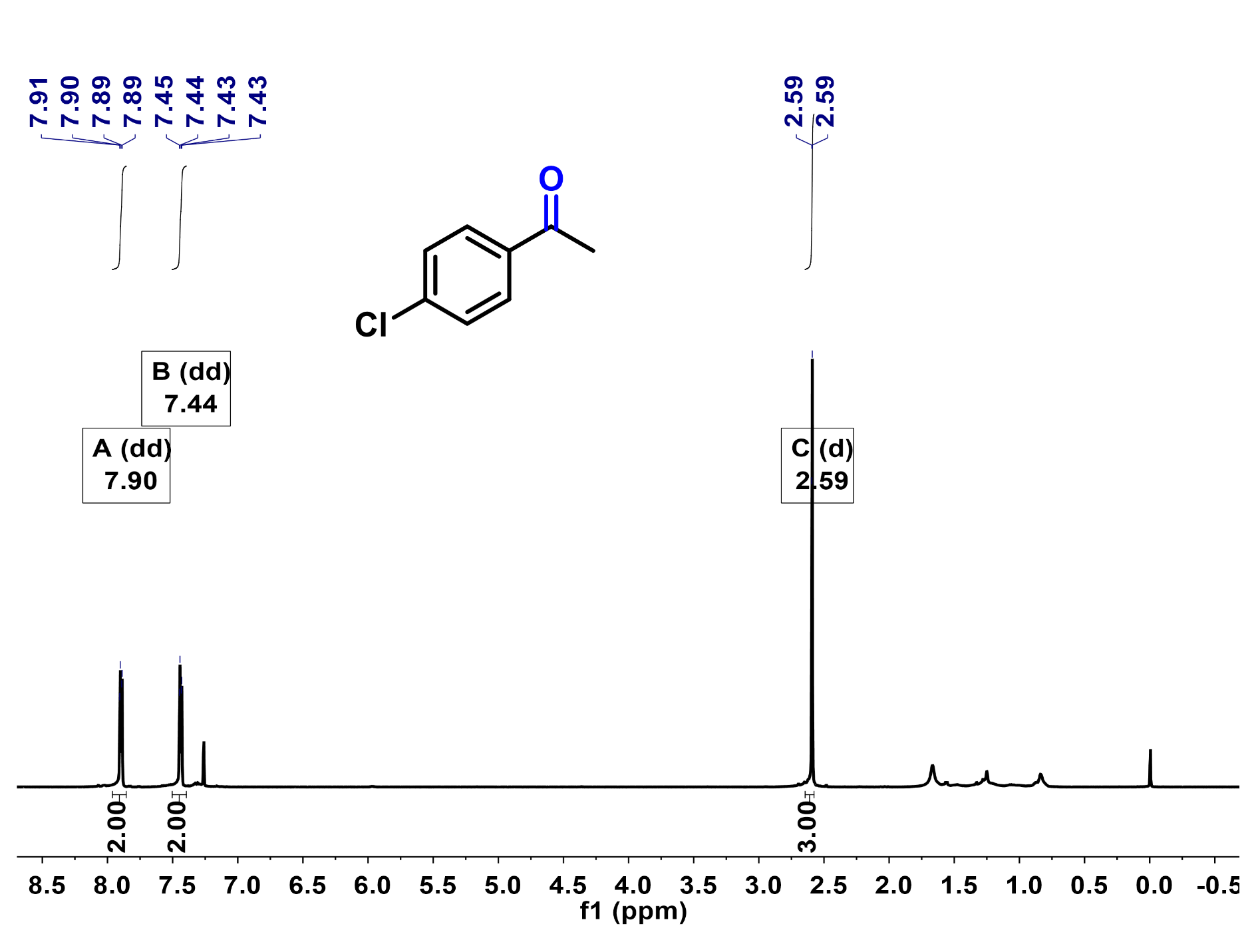


**Fig. S9.** ^1^H NMR spectrum (600 MHz, CDCl_3_) of 4'-chloroacetophenone after TLC separation for purification.


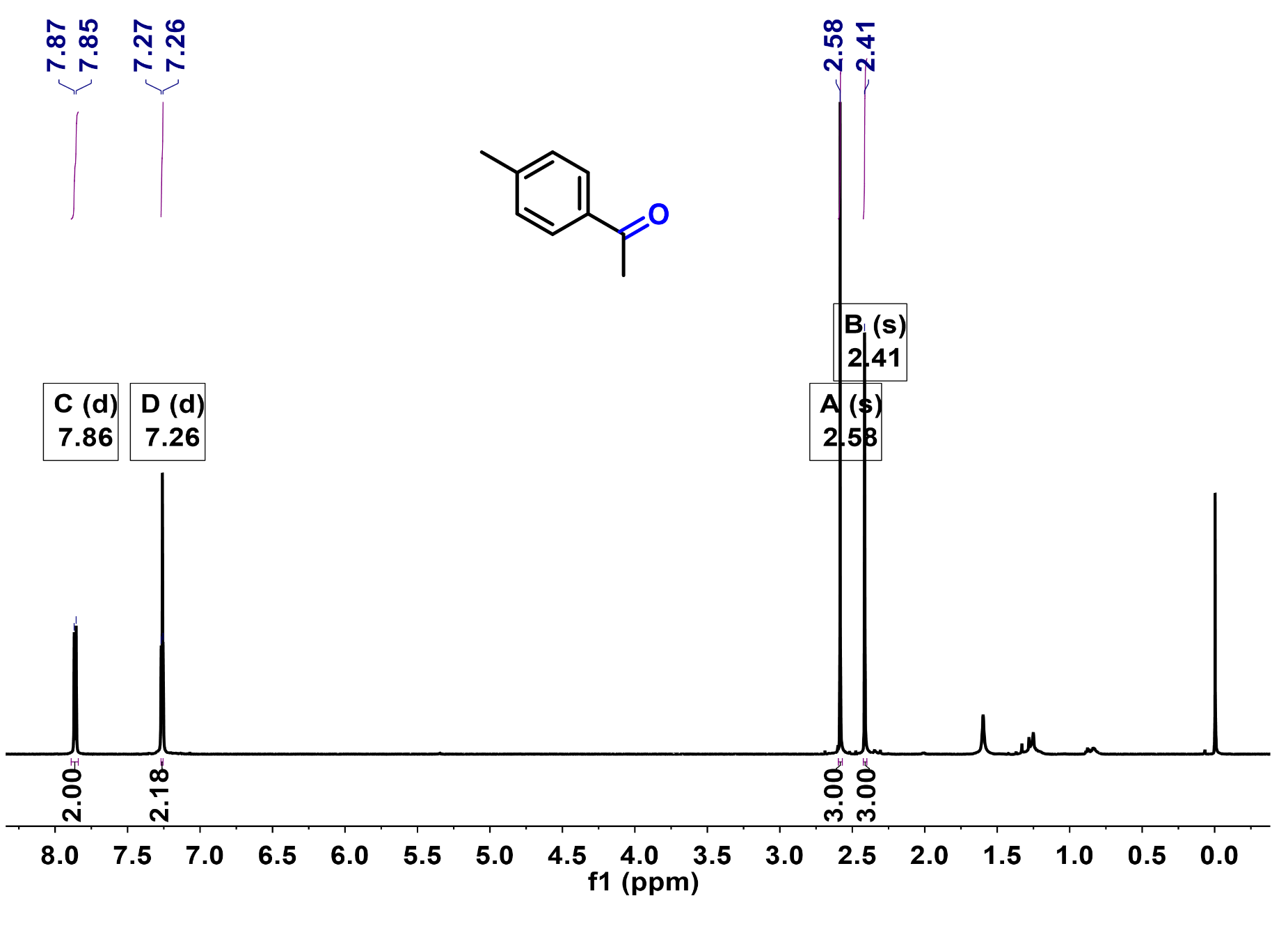


**Fig. S10.** ^1^H NMR spectrum (600 MHz, CDCl_3_) of 4'-methylacetophenone after TLC separation for purification.


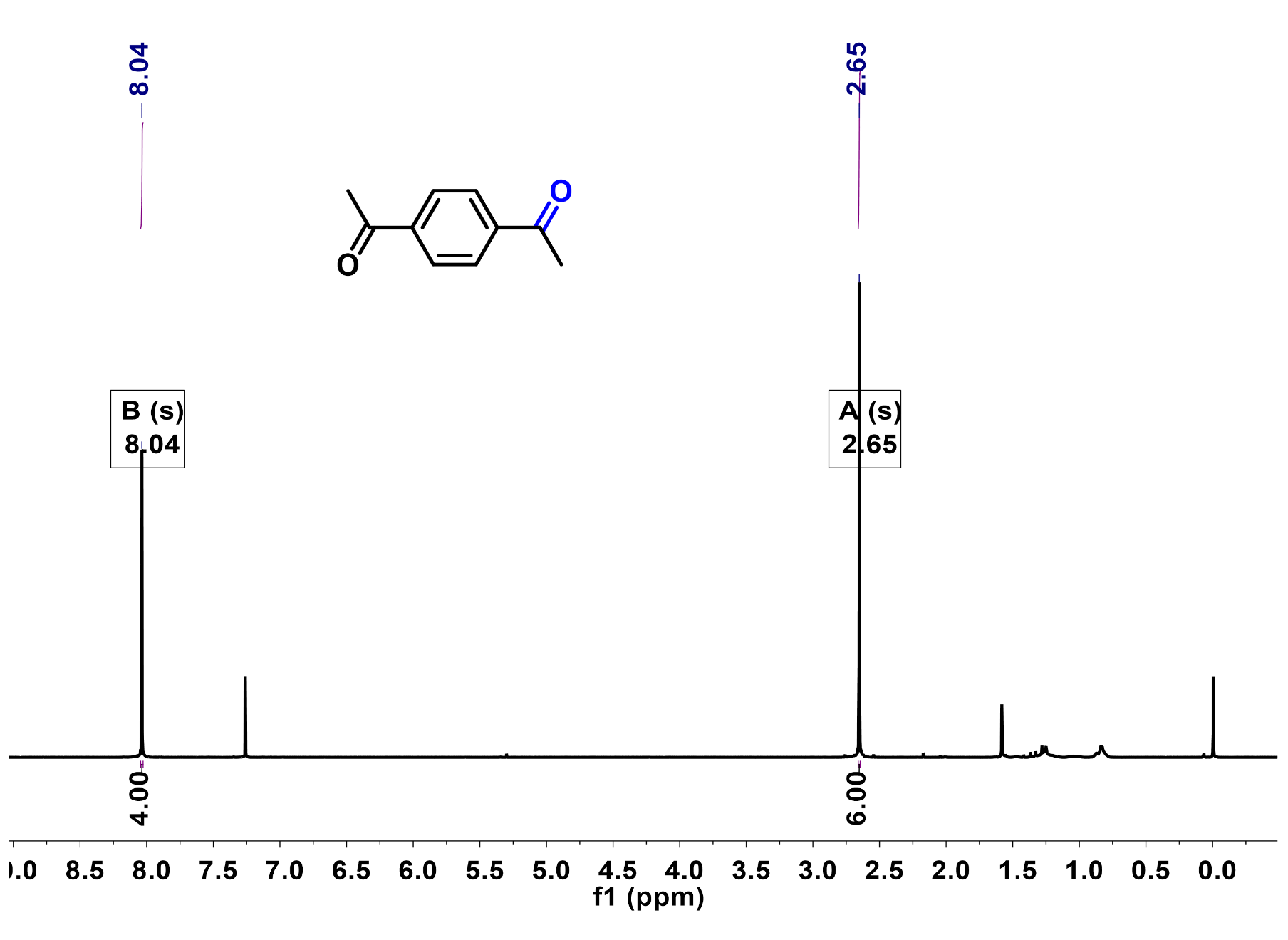


**Fig. S11.** ^1^H NMR spectrum (600 MHz, CDCl_3_) of 1,4-diacetylbenzene after TLC separation for purification.


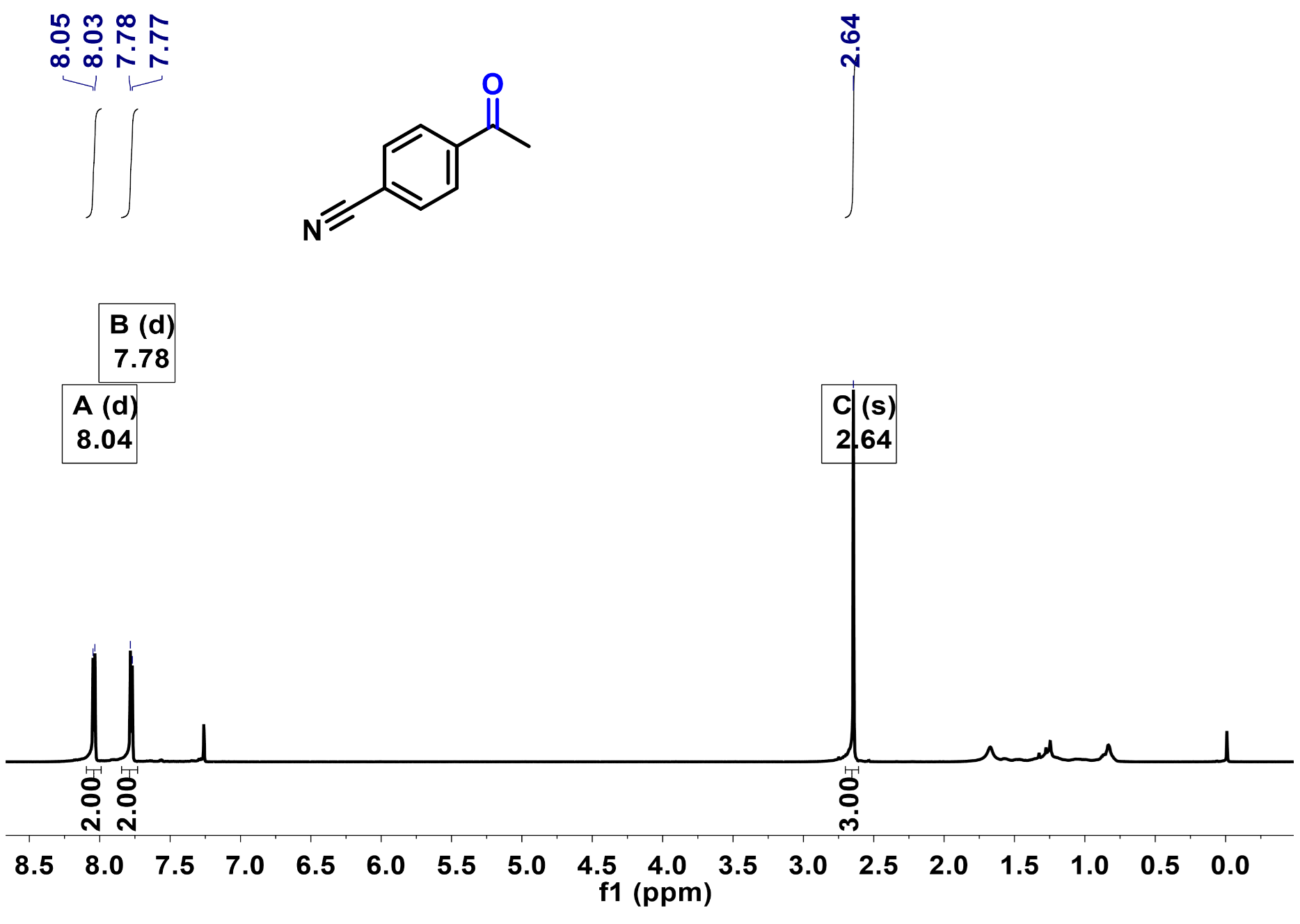


**Fig. S12.** ^1^H NMR spectrum (600 MHz, CDCl_3_) of 4-acetylbenzonitrile after TLC separation for purification.


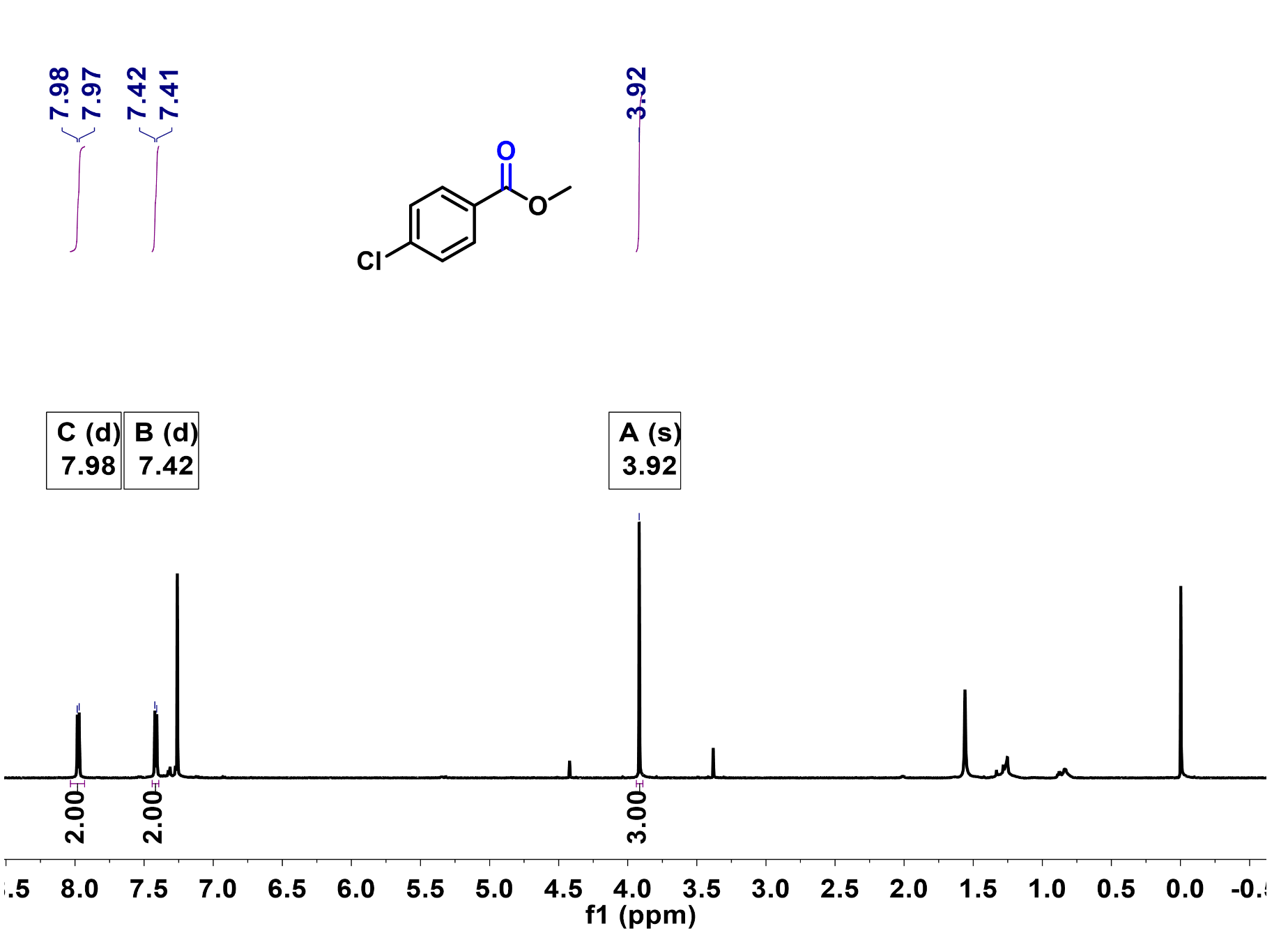


**Fig. S13.** ^1^H NMR spectrum (600 MHz, CDCl_3_) of methyl 4-chlorobenzoate after TLC separation for purification.


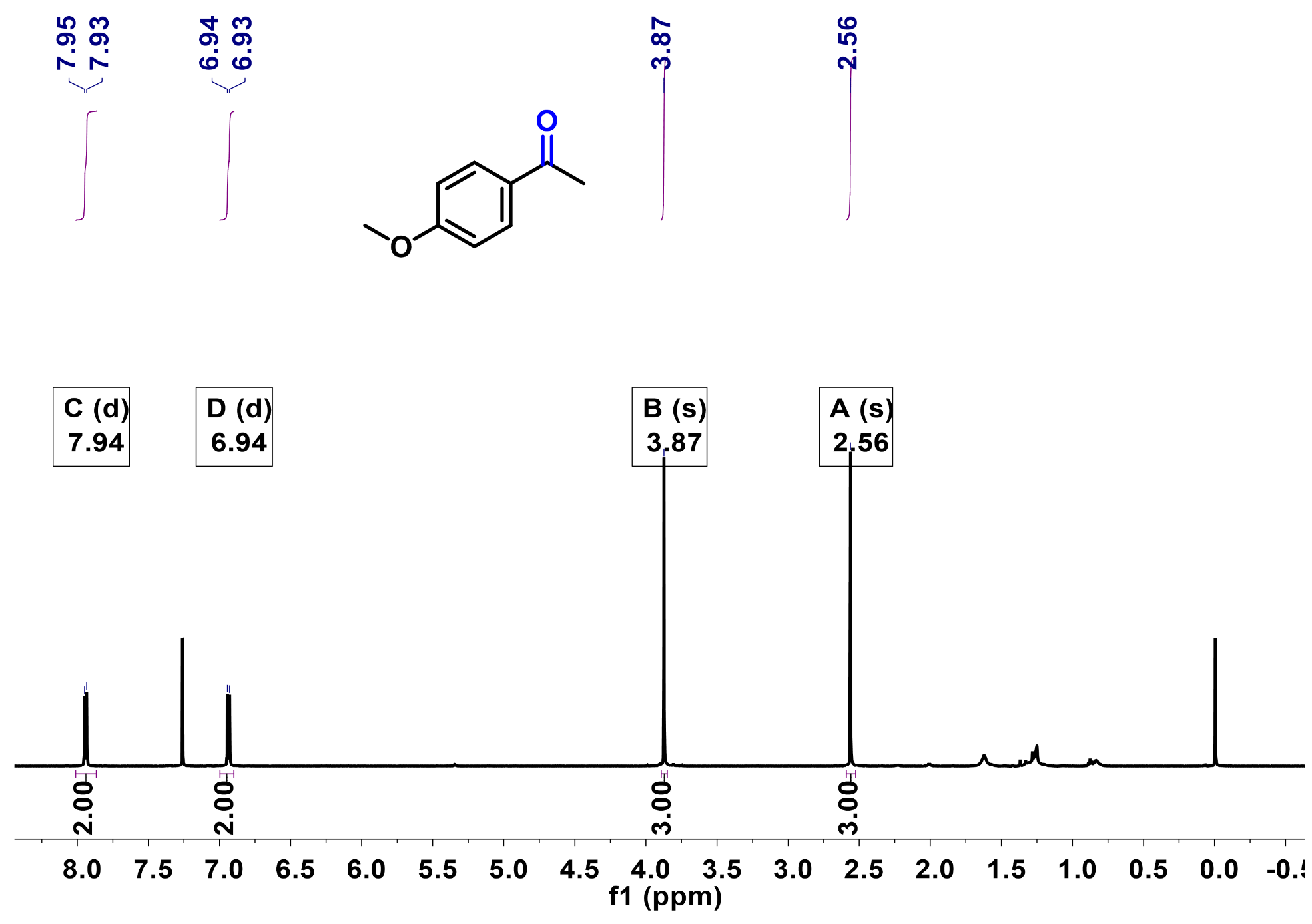


**Fig. S14.** ^1^H NMR spectrum (600 MHz, CDCl_3_) of 4'-methoxyacetophenone after TLC separation for purification.


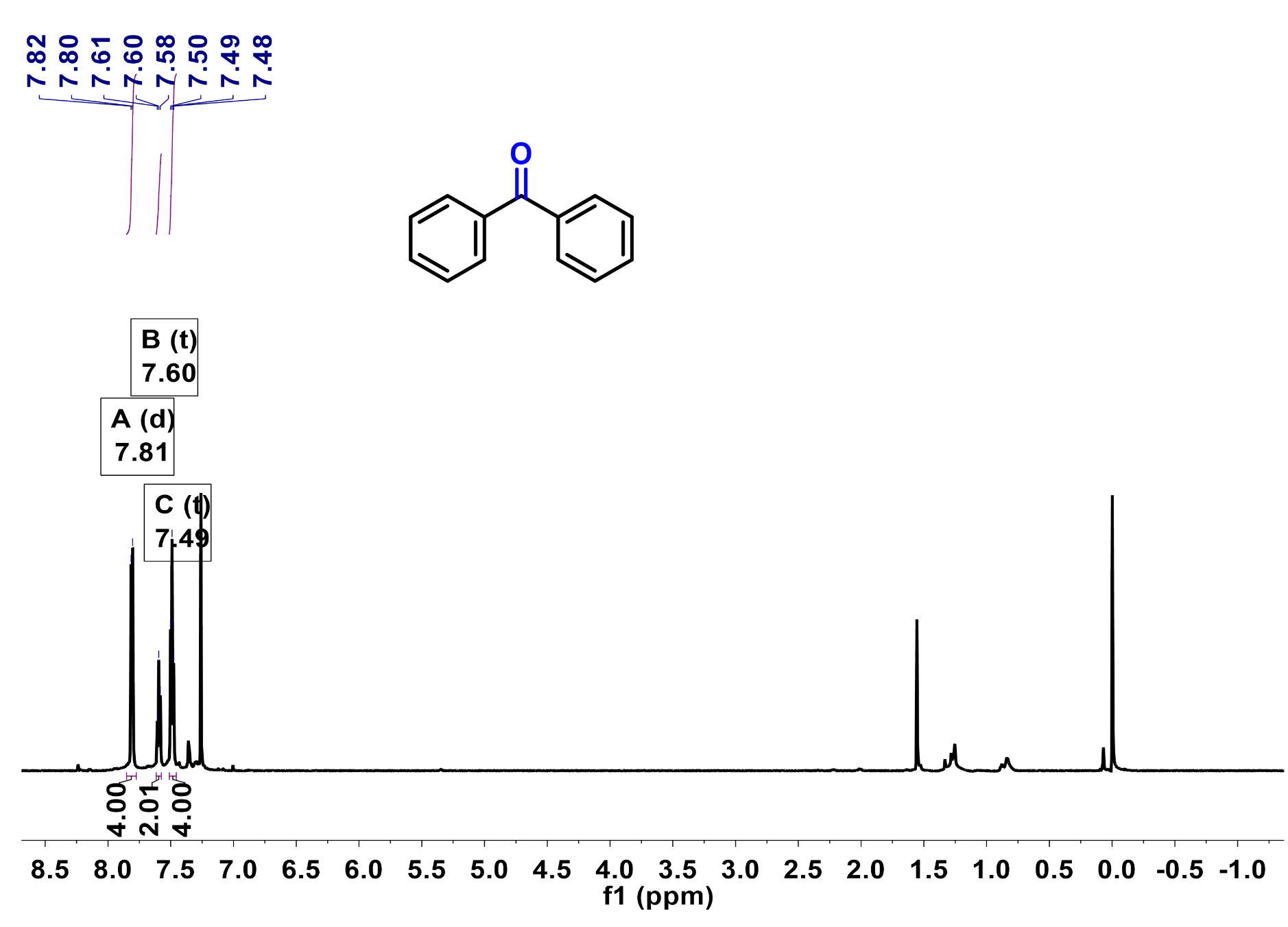


**Fig. S15.** ^1^H NMR spectrum (600 MHz, CDCl_3_) of benzophenone after TLC separation for purification.


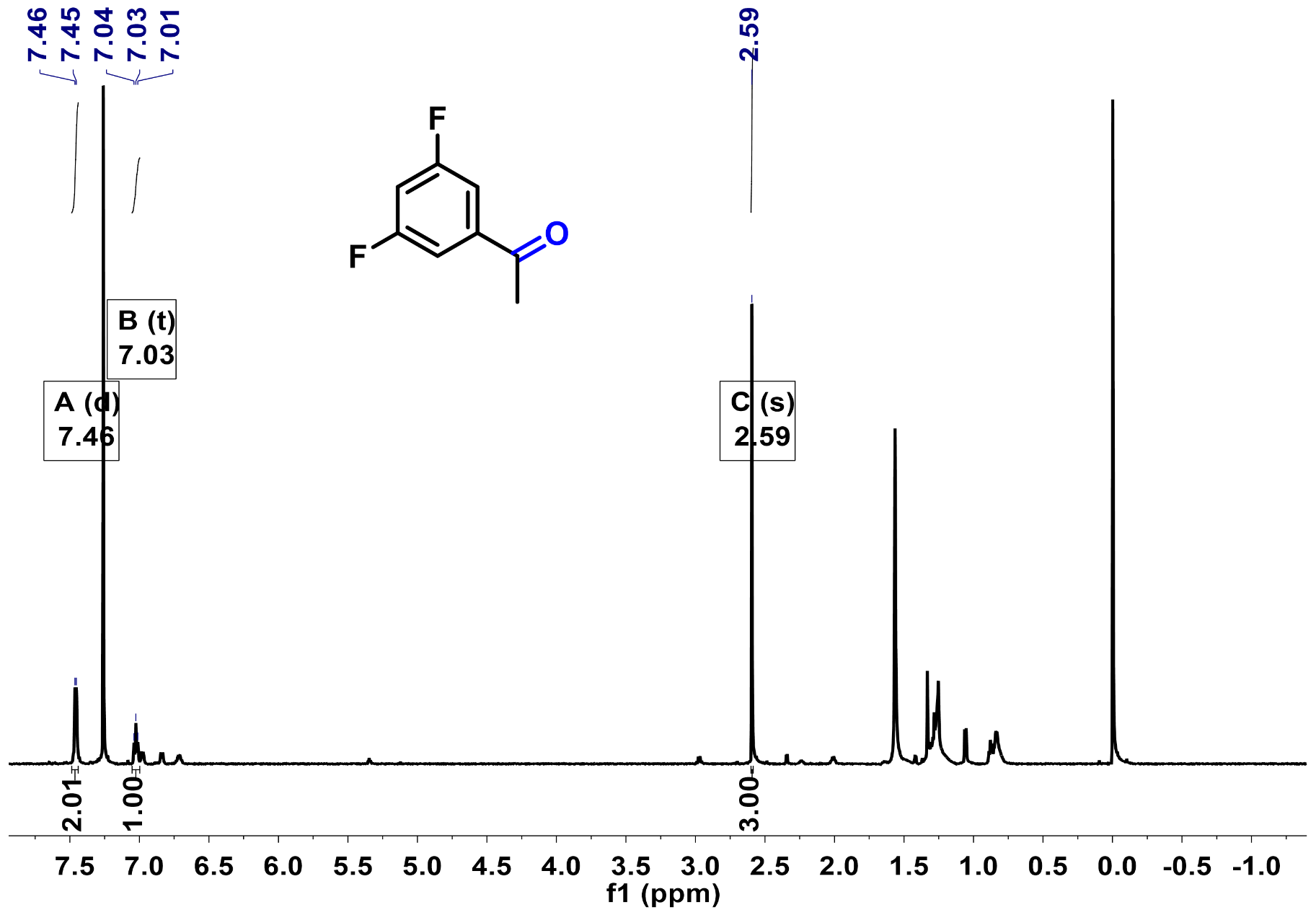


**Fig. S16.** ^1^H NMR spectrum (600 MHz, CDCl_3_) of 1-(3,5-difluorophenyl)ethan-1-one after TLC separation for purification.


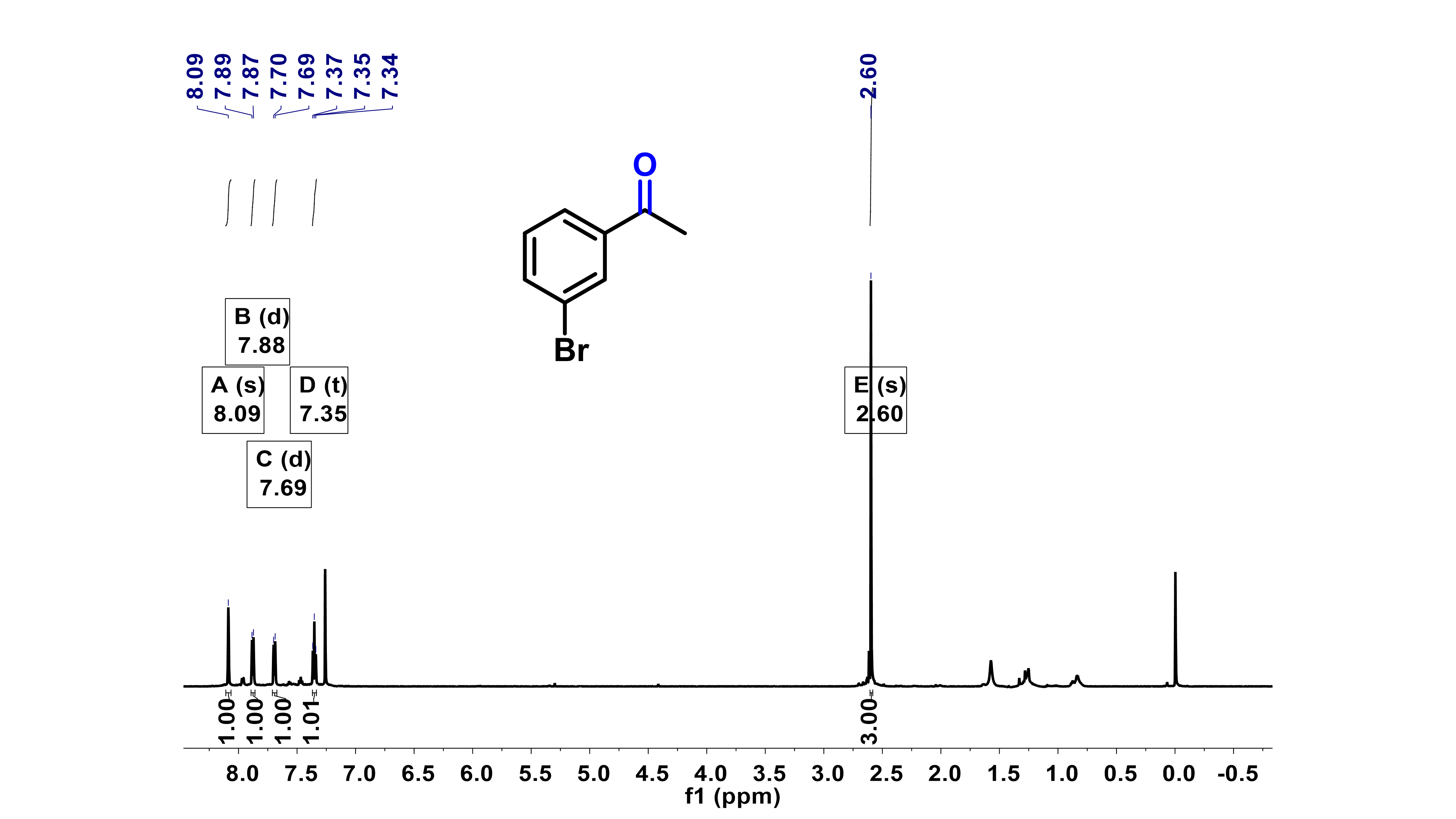


**Fig. S17.** ^1^H NMR spectrum (600 MHz, CDCl_3_) of 1-(3-bromophenyl)ethan-1-one after TLC separation for purification.


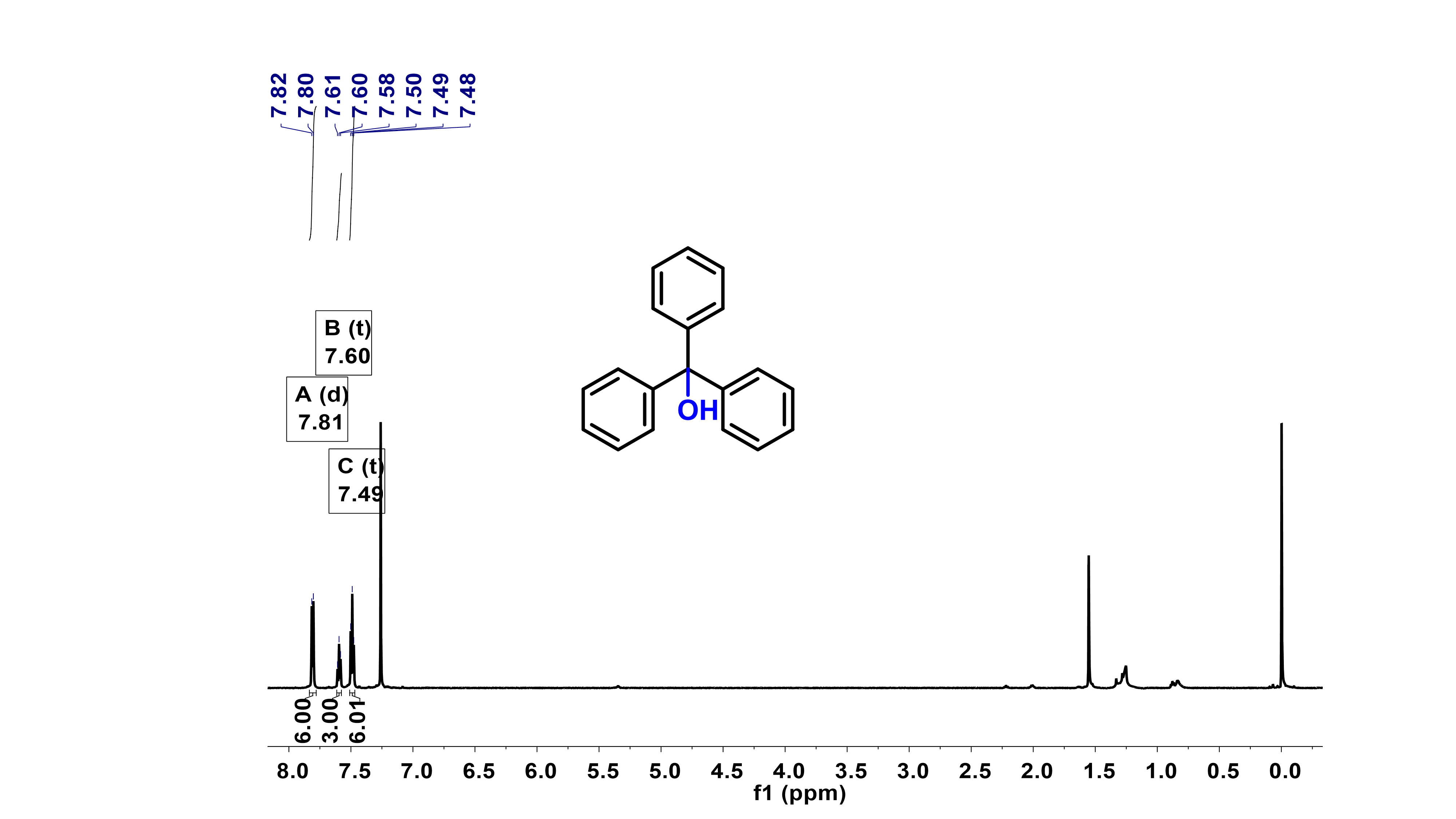


**Fig. S18.** ^1^H NMR spectrum (600 MHz, CDCl_3_) of triphenylmethanol after TLC separation for purification.


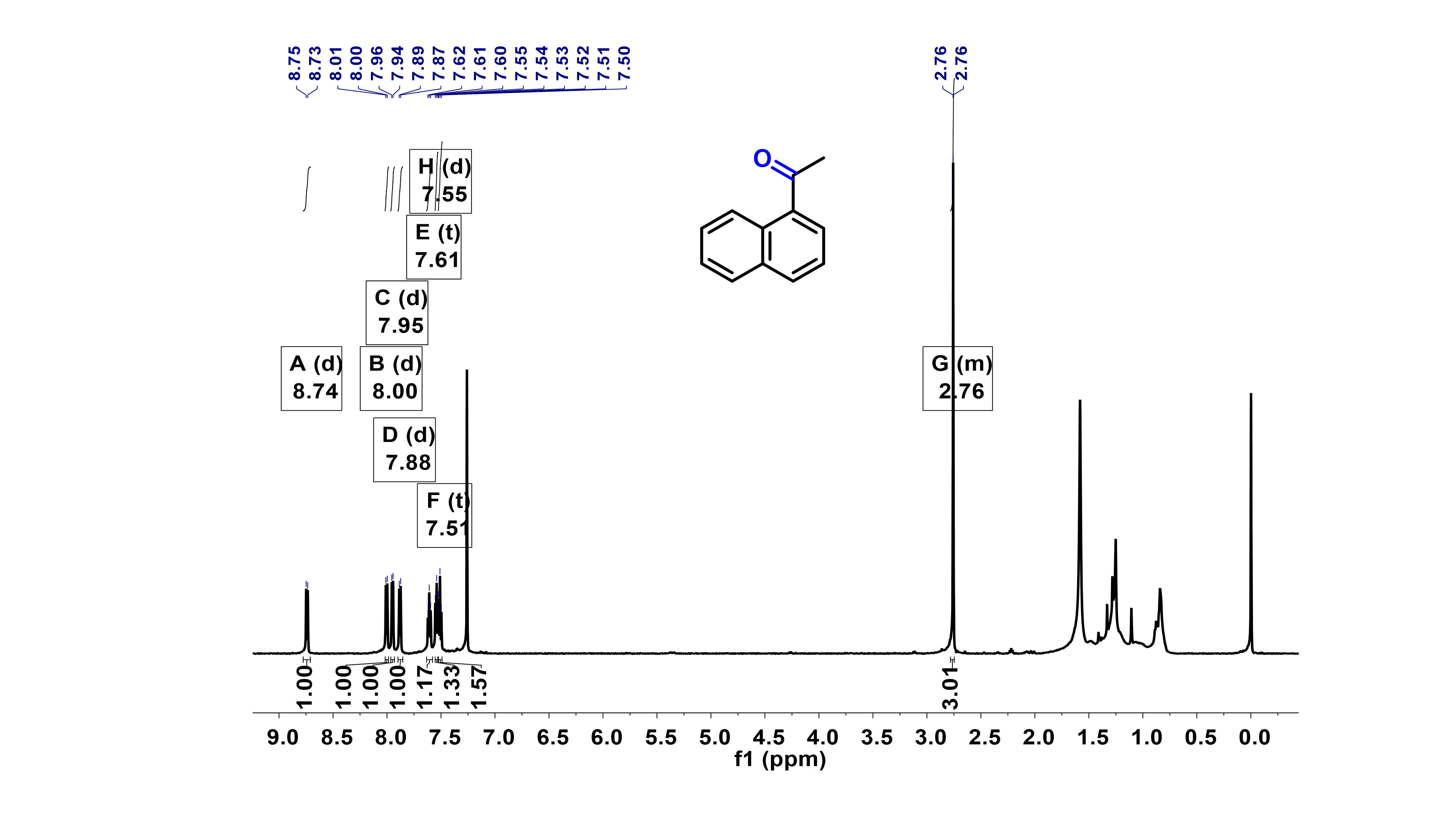


**Fig. S19.** ^1^H NMR spectrum (600 MHz, CDCl_3_) of 1-(naphthalen-1-yl)ethan-1-one after TLC separation for purification.


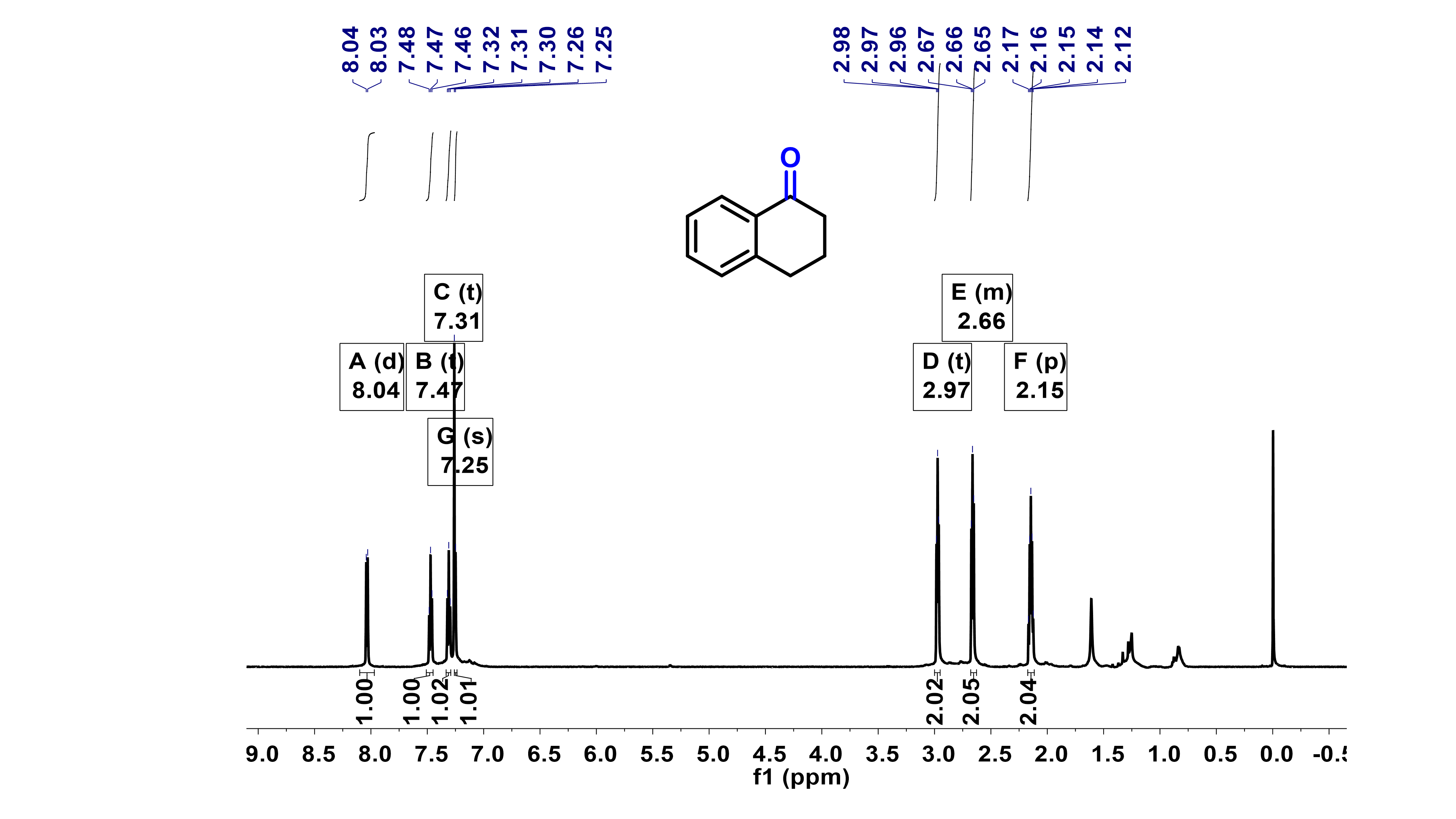


**Fig. S20.** ^1^H NMR spectrum (600 MHz, CDCl_3_) of 3,4-dihydronaphthalen-1(2H)-one after TLC separation for purification.


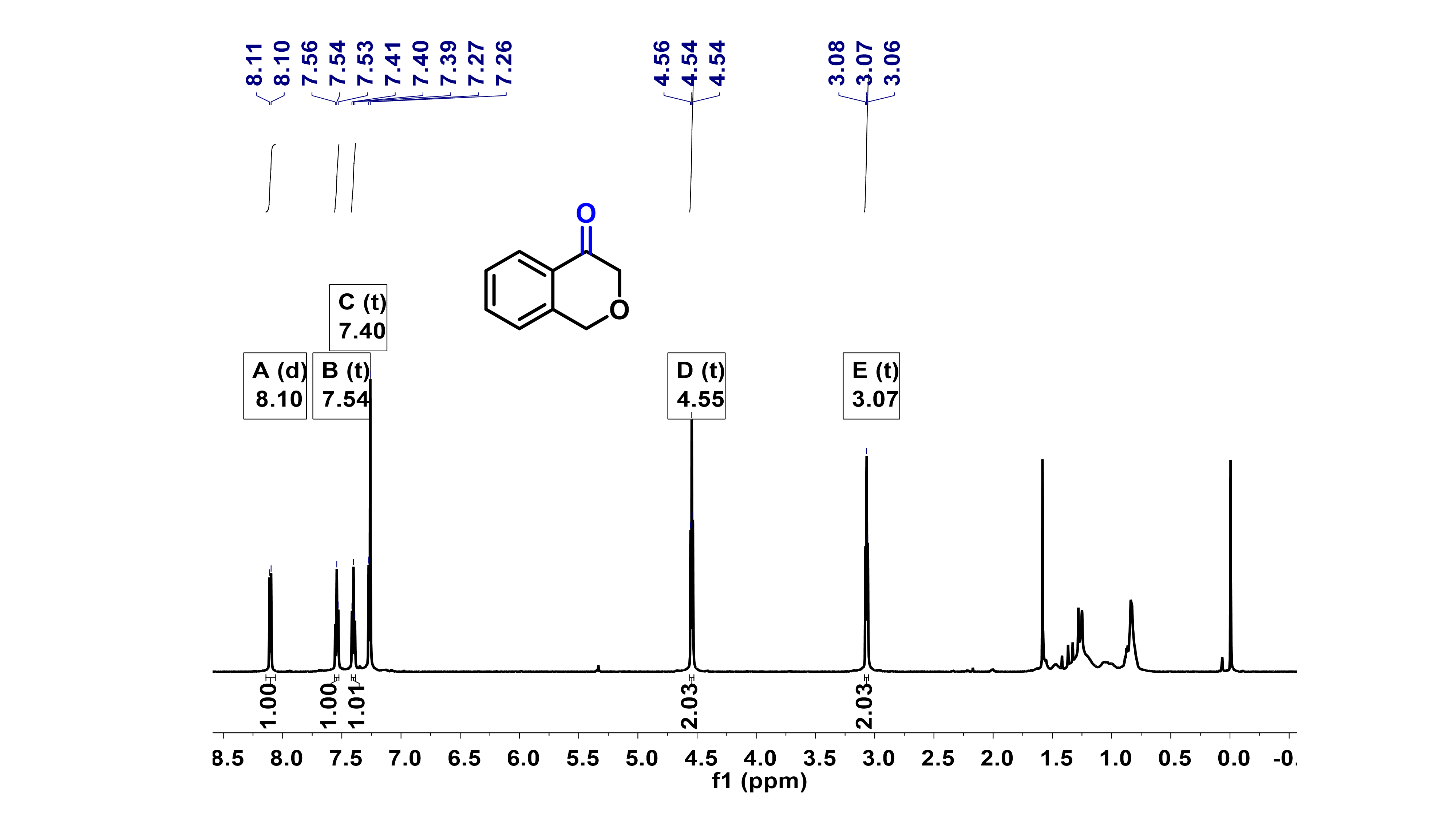


**Fig. S21.** ^1^H NMR spectrum (600 MHz, CDCl_3_) of isochroman-4-one after TLC separation for purification.


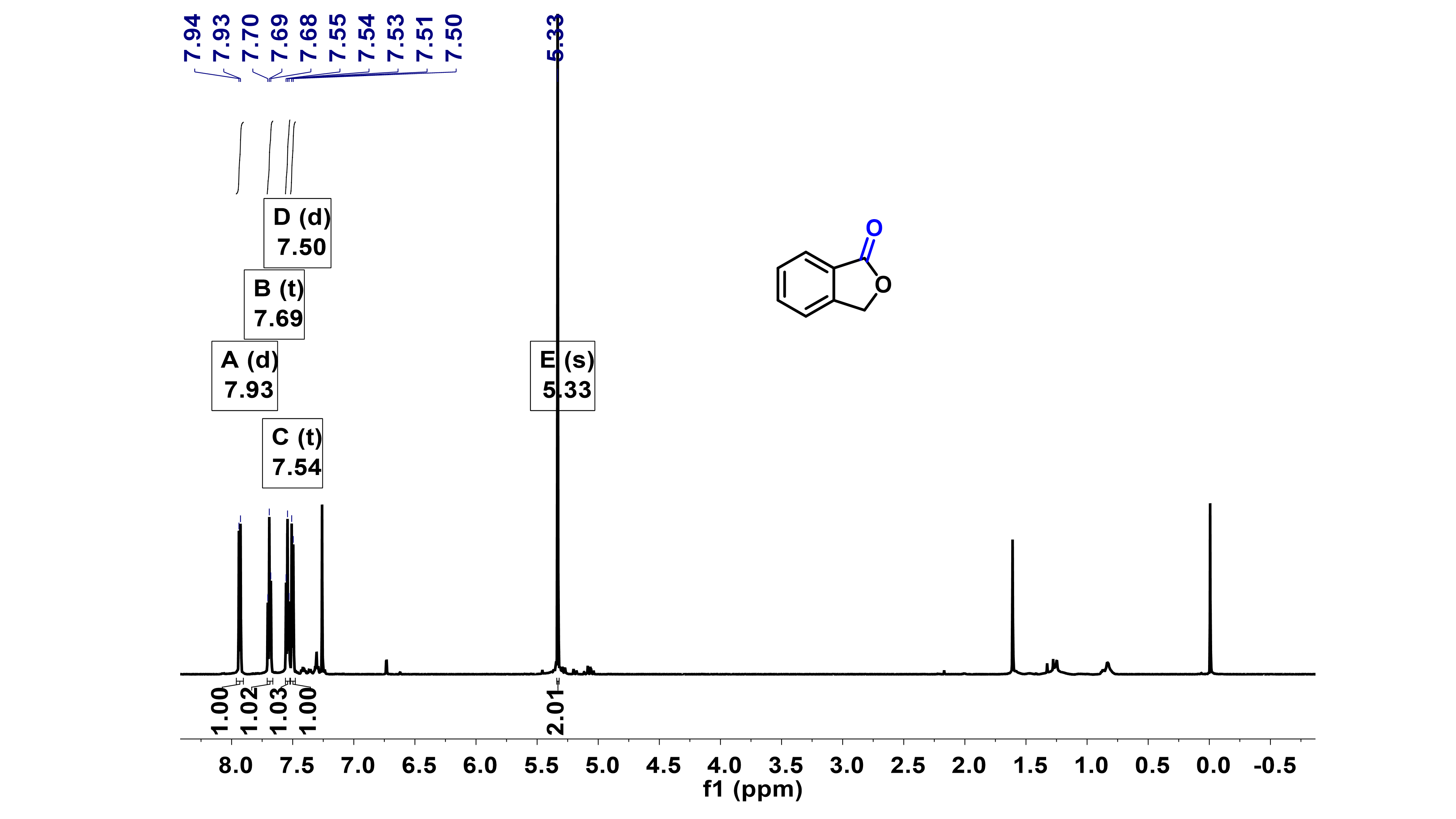


**Fig. S22.** ^1^H NMR spectrum (600 MHz, CDCl_3_) of isobenzofuran-1(3H)-one after TLC separation for purification.


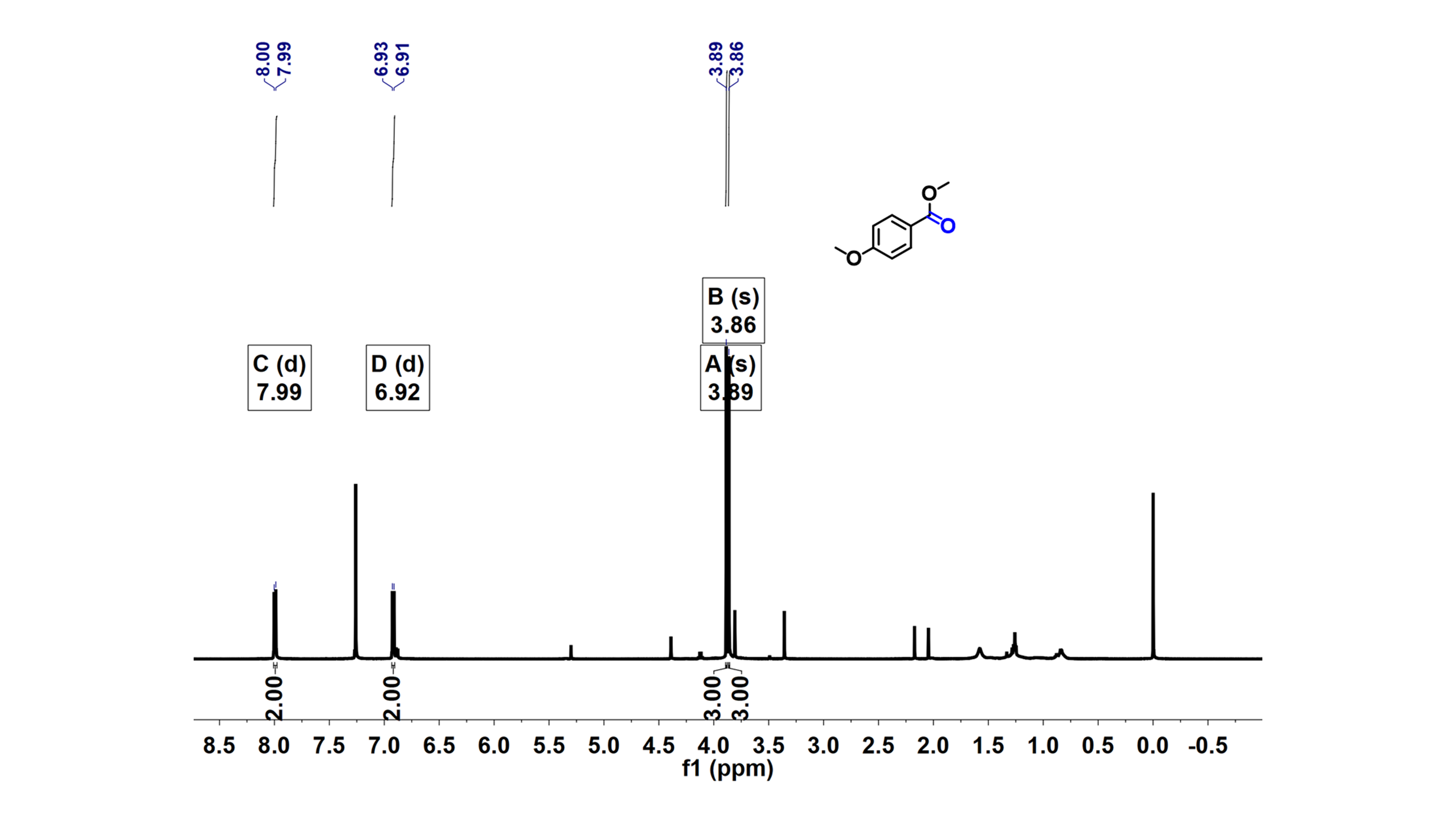


**Fig. S23.** ^1^H NMR spectrum (600 MHz, CDCl_3_) of methyl 4-methoxybenzoate after TLC separation for purification.


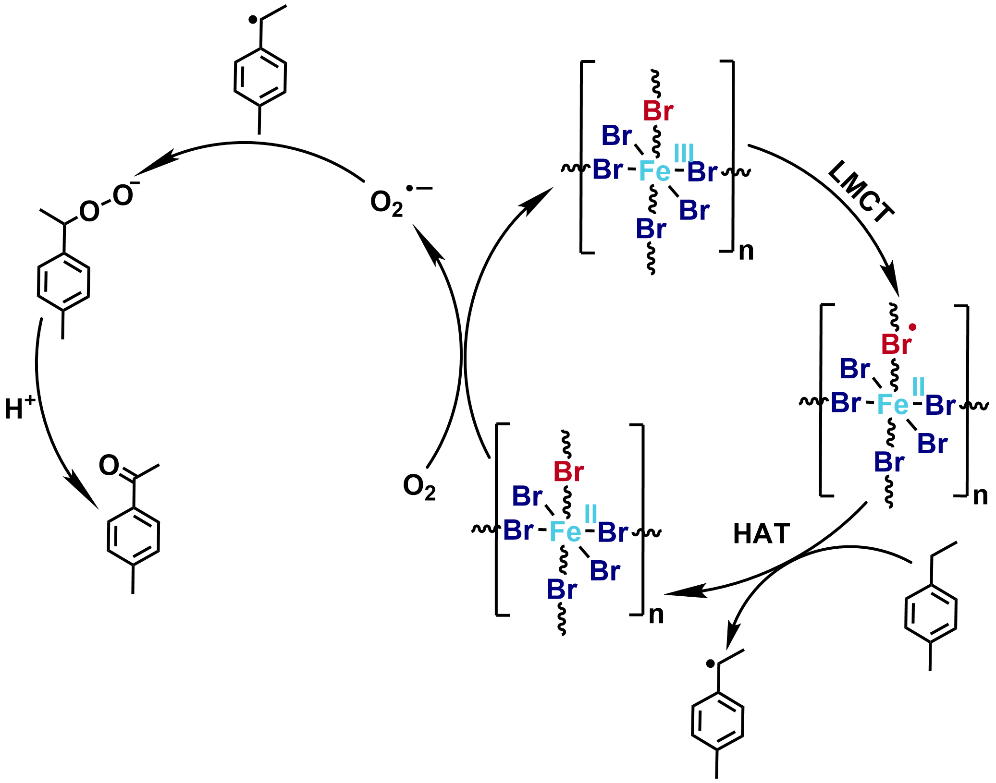


**Fig. S24.** Diagram illustrating the ferroelectric catalytic cycle mechanism of DEFM-FeBr_4_.





**Fig. S25.** Electrospray ionization-high-resolution mass spectrometry (ESI-HRMS) spectrum of the C_7_H_14_^•^ radical adduct with TEMPO.





**Fig. S26.** Electron paramagnetic resonance (EPR) spectra of DEFM-FeBr_4_ suspensions containing 5,5-dimethyl-1-pyrroline-N-oxide (DMPO) (0.1 mM) in the dark (black line), under 365 nm LED irradiation (blue line), dark + ultrasound conditions (green line) and under light + ultrasound conditions (red line).
